# Effects of anion complexation on the photoreactivity of bisureido- and bisthioureido-substituted dibenzobarrelene derivatives

**DOI:** 10.3762/bjoc.7.37

**Published:** 2011-03-04

**Authors:** Heiko Ihmels, Jia Luo

**Affiliations:** 1 Organic Chemistry II, University of Siegen, Adolf-Reichwein-Str. 2, D-57068 Siegen, Germany

**Keywords:** dibenzobarrelenes, dibenzosemibullvalenes, di-π-methane rearrangement, supramolecular photochemistry, (thio)urea derivatives

## Abstract

Bisureido- and a bisthioureido-substituted dibenzobarrelene derivative were synthesized and the photoreactivity of two representative examples were studied. Direct irradiation of the ureido-substituted derivative induces a di-π-methane rearrangement to the corresponding dibenzosemibullvalene derivative, whereas the thioureido-substituted derivative is almost photoinert. Complexes of the latter derivative with chloride, carboxylates, or sulfonate anions, however, are efficiently transformed to the dibenzosemibullvalene product upon irradiation, presumably by suppressing the self-quenching of the thiourea units in the complex. The association of the ureido-substituted dibenzobarrelene derivative with (*S*)-mandelate and irradiation of this complex led to the formation of the dibenzosemibullvalene with moderate stereoselectivity (68:32 er). In contrast, the thioureido derivative showed no such effect upon complexation of chiral anions.

## Introduction

The control of the selectivity of a photoreaction by supramolecular interactions has recently received much attention [1–3]. For example, chiral receptors have been employed that associate with photoreactive substrates, leading to a distinct preferential conformation of the latter and/or to a limited exposure of the substrate to other reagents due to the shielding effect of the receptor. Because of the restricted freedom of movement or availability of reactive sites within this assembly, mono- and bimolecular photoreactions may proceed through one preferential pathway resulting in regio- or stereoselective product formation. Indeed, this approach has been employed to carry out stereoselective photoreactions, for example [2 + 2] cycloaddition [4], [4 + 4] photocycloaddition [5], Norrish–Yang cyclization [6], and [6π] photocyclization [7]. Asymmetric photoreactions have also been carried out with very good stereoselectivities in organized or constrained media [8–10]. For example, photoactive substrates may be accommodated as guest molecules in chiral host systems, such as suitably modified cucurbiturils [11–15], self-assembled cages [16] and bowls [17], liquid crystals [18], chiral crystals [19–23], or cyclodextrins (CDs) [24–26] in such a way that the chiral environment within the binding site has an influence on preferential reaction pathways, thus inducing stereoselective photoreactions.

**Scheme 1:**
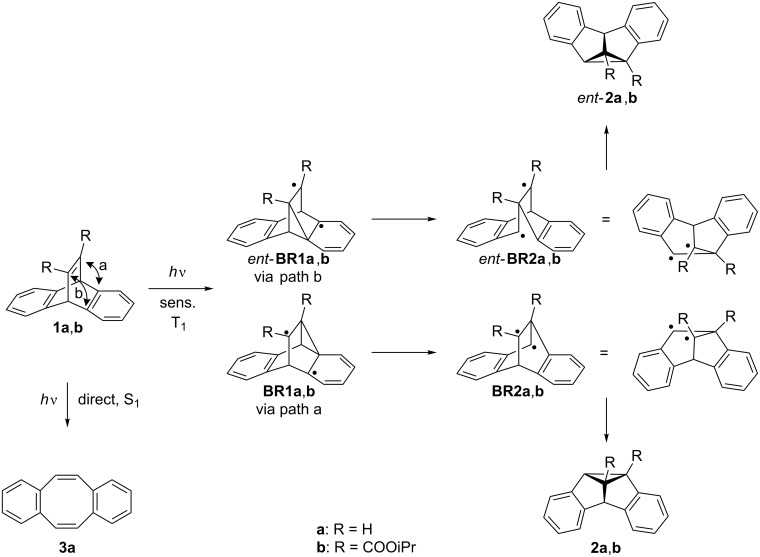
Photorearrangements of dibenzobarrelenes **1a** and **1b**.

Along these lines, the di-π-methane (DPM) rearrangement [27–28] of dibenzobarrelene (dibenzobicyclo[2.2.2]octatriene) (**1a**) and its derivatives has been shown to be an appropriate model reaction for the assessment of substituent effects on the selectivity of organic photoreactions (Scheme 1) [29–30]. The photoreactivity of dibenzobarrelene derivatives is multiplicity-dependent: The direct irradiation of **1a** leads to the dibenzocyclooctatetraene **3a** in a singlet reaction that occurs via an initial [2 + 2] cycloaddition followed by a [4 + 2] retro-Diels–Alder reaction [27–30]. In the presence of a triplet sensitizer, e.g., acetone or benzophenone, a triplet-state di-π-methane rearrangement is induced. Thus, in the initial reaction step connection between one vinyl and one benzo carbon atom takes place, i.e., a so called vinyl–benzo bridging, that leads to the intermediate biradical **BR1a** [29]. Subsequent rearomatization with the formation of the intermediate **BR2a** and intramolecular radical recombination gives the dibenzosemibullvalene **2a** as the reaction product. Notably, the DPM rearrangement of dibenzobarrelene derivatives such as **1b**, that carry substituents other than hydrogen atoms at the vinyl positions, leads to the formation of two enantiomeric dibenzosemibullvalenes **2b** and *ent*-**2b**. As indicated in Scheme 1, the two enantiomers originate from different vinyl–benzo bridging pathways in the first reaction step (path a or b). Note that the same two enantiomers are formed upon initial vinyl–benzo bridging with the other benzene unit.

**Scheme 2:**
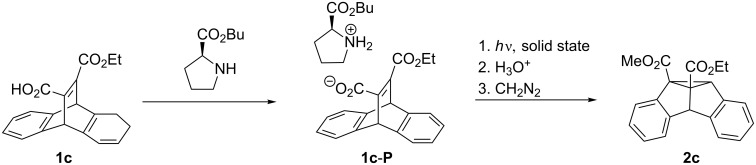
Stereoselective DPM rearrangement of chiral salts in the solid-state.

Stereoselective DPM rearrangements of dibenzobarrelene derivatives have been reported in special media, such as chiral mesoporous silica [31] or ionic-liquids [32]; however, most examples for stereoselective DPM rearrangements of dibenzobarrelene derivatives have been observed in the solid-state. For example, the achiral derivative **1b** crystallizes in the chiral space group *P*2_1_2_1_2_1_, and irradiation of the chiral crystals gives dibenzosemibullvalene **2b** with a high enantiomeric excess, >95% ee [33]. Since achiral molecules crystallize only very rarely in chiral space groups, the ionic chiral auxiliary strategy was developed by Scheffer et al. which allows to influence the stereoselectivity of solid-state photoreactions by chiral counter ions [34]. This is accomplished by providing the chromophore under investigation with a carboxylic acid functionality and then by attaching a chiral, enantiomerically pure amine by salt formation. An optically active salt is obtained, which consequently crystallizes in a chiral space group. The irradiation of these salts in the solid-state leads to enantiomerically enriched photoproducts. This approach has been successfully applied to the carboxy-substituted dibenzobarrelene derivative **1c** which forms the chiral ammonium carboxylate **1c-P** with (*S*)-proline (Scheme 2). After irradiation, acidic workup and subsequent esterification with diazomethane, the dibenzosemibullvalene **2c** was obtained with high enantiomeric excess (>95% ee) [35–36].

Interestingly, several asymmetric photoreactions have been conducted with remarkable enantioselectivity in homogeneous solution, whereas reports of asymmetric di-π-methane rearrangements in solutions are relatively rare. Chiral auxiliaries attached as ester or amide functionalities at the vinylic positions of dibenzobarrelene induce only low enantioselectivities in the DPM rearrangement in solution [37]; and the ionic auxiliary strategy, which generates impressive enantioselectivity in the solid-state, fails to induce any stereoselectivity in DPM rearrangements in solution. Considering these observations, it remains a challenge to develop a method to accomplish stereoselective DPM rearrangements of dibenzobarrelene derivatives in homogenous solutions. Therefore, we intended to study whether supramolecular interactions of chiral additives with achiral dibenzobarrelenes may be used to influence the photoreactivity of the latter in solution. For that purpose the dibenzobarrelene chromophore was functionalized with ureido or thioureido substituents, since these functionalities are strong hydrogen bonding donors, which may associate with (chiral) anions [38–39]. Moreover, the versatile use of urea and thiourea derivatives in organocatalysis has been demonstrated in several examples [40–44]. Herein, we report the synthesis of ureido- and thioureido-substituted dibenzobarrelene derivatives **1e–i**, along with first studies of their photochemical properties in the absence and in the presence of anions.

## Results

**Scheme 3:**
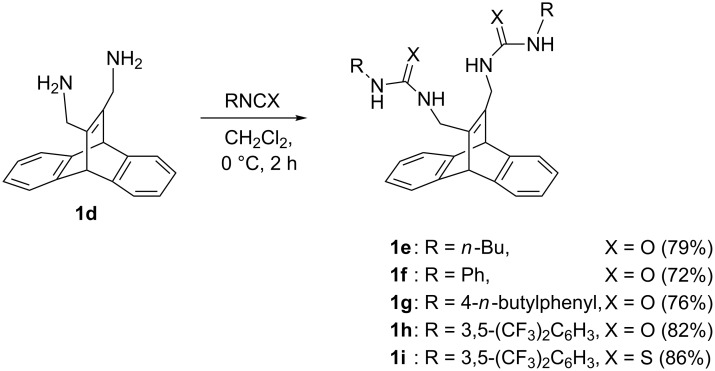
Synthesis of ureido- and thioureido-substituted dibenzobarrelene derivatives **1e**–**i**.

The bisureido- and bisthioureido-substituted dibenzobarrelene derivatives **1e–i** were synthesized by the reaction of the known bis(diaminomethyl)-substituted derivative of dibenzobarrelene **1d** [45] with a slight excess of the corresponding isocyanate or isothiocyanate (Scheme 3). The resulting products precipitated from the reaction mixture and were isolated in good yields (72–86%) by direct filtration, except for the thioureido-substituted derivative **1i**, which was crystallized from ethyl acetate to give crystals containing one molecule of ethyl acetate as indicated by ^1^H NMR spectroscopy and elemental analysis. All products were fully characterized by ^1^H and ^13^C NMR spectroscopy, mass spectrometry, and elemental analysis. The solubility of the dibenzobarrelene derivatives **1e–g** is very low in most organic solvents (e.g., <5 mg/l in acetonitrile at 20 °C). In contrast, the 3,5-bis(trifluoromethyl)phenyl-substituted derivatives **1h** and **1i** have significantly improved solubility in organic solvents; i.e., compound **1h** has good solubility in acetone, acetonitrile and alcohols, while thiourea **1i** dissolves in most polar organic solvents.

**Scheme 4:**
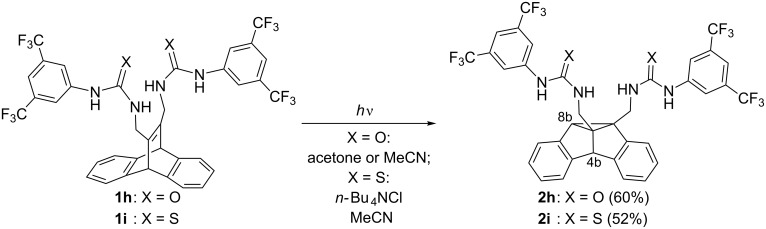
Di-π-methane rearrangements of ureido- and thioureido-substituted dibenzobarrelene derivatives **1h** and **1i**.

Because of their favorable solubility in organic solvents, the dibenzobarrelene derivatives **1h** and **1i** were used for the systematic photochemical studies. In both acetone and acetonitrile, irradiation of the bisureido-substituted derivative **1h** gave the dibenzosemibullvalene **2h** as the major photoproduct (Scheme 4). After irradiation of dibenzobarrelene **1h** in acetone solution, product **2h** was isolated in 60% yield by crystallization directly from the reaction mixture. The structural assignment of **2h** was based on the characteristic ^1^H NMR shifts of the two singlets for the protons at C*8b* (3.22 ppm) and C*4b* (4.63 ppm). Irradiation of compound **1h** through a quartz filter (λ > 254 nm) resulted in rapid conversion of **1h**. The photolysate contained ca. 60% of **2h**, as determined by ^1^H NMR spectroscopic analysis. The byproducts could not be identified. In contrast, no byproducts were formed when the irradiation was carried out through Duran glass (λ > 310 nm); however, in this case a longer irradiation time was required . For example, after irradiation of a solution of **1h** in acetonitrile (10^−3^ M) through Duran glass for 8 h, TLC analysis still indicated the presence of the starting material, whereas in acetone solution full conversion was observed after 3 h irradiation under otherwise identical conditions. The reaction was significantly faster in the presence of anions: The irradiation of a solution of **1h** and two molar equiv of tetrabutylammonium chloride (TBAC) in acetonitrile for 3 h (10^−3^ M, λ > 310 nm) led to complete conversion, and the semibullvalene **2h** was obtained in 84% yield after column chromatography. Similar results were obtained in the presence of carboxylate or sulfonate salts.

The irradiation of the bisthioureido-substituted dibenzobarrelene derivative **1i** in various organic solvents did not induce the DPM rearrangement, even in acetone, as indicated by TLC and ^1^H NMR spectroscopic analysis of the reaction mixture. Instead, ^1^H NMR spectroscopic analysis revealed slow decomposition of the dibenzobarrelene derivative **1i** upon irradiation with no formation of distinct photoproducts. In contrast, the irradiation of compound **1i** in the presence of 2 molar equiv of either tetrabutylammonium chloride (TBAC) or tetrabutylammonium (*S*)*-*camphor-10-sulfonate (**SCS**) in acetonitrile for 4–6 h converted the dibenzobarrelene **1i** into the dibenzosemibullvalene **2i** (Scheme 4), as indicated by the characteristic singlets of the dibenzosemibullvalene structure in the ^1^H NMR spectrum (*8b-*H: 3.43 ppm; *4b*-H: 4.76 ppm, in acetone). The dibenzosemibullvalene **2i** was obtained in 52% yield by filtration through a column of silica gel and subsequent crystallization. The dibenzosemibullvalenes **2h** and **2i** were identified and fully characterized by ^1^H NMR and ^13^C NMR spectroscopy, elemental analysis and/or mass spectrometry.

**Figure 1:**
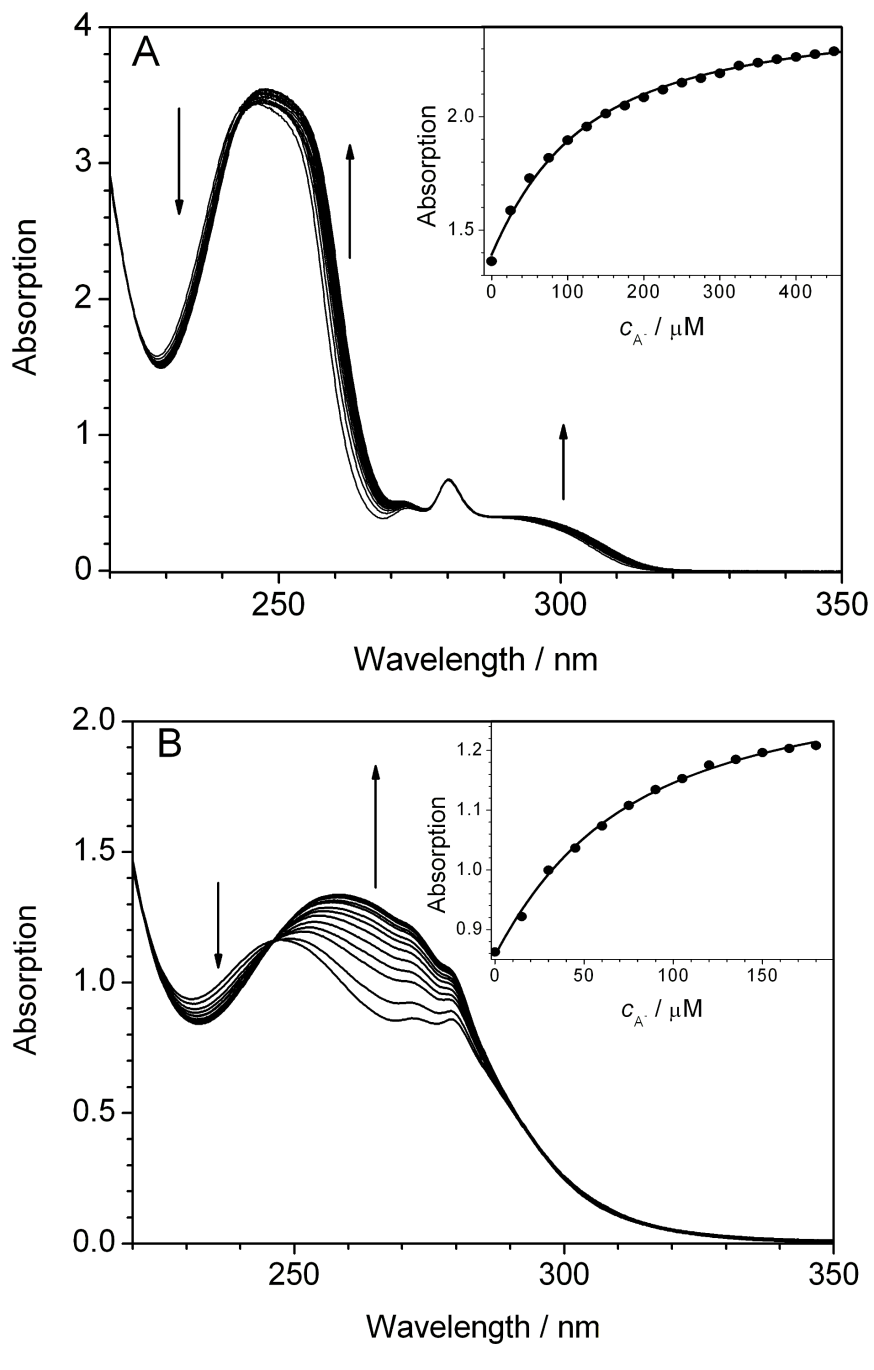
Photometric titration of A) tetrabutylammonium chloride (TBAC) to **1h** (*c***_1h_** = 50 µM) and of B) tetrabutylammonium (10*S*)*-*camphorsulfonate (**SCS**) to **1i** (*c***_1i_** = 30 µM). Arrows indicate the changes of the absorption with increasing concentration of the salt. Inset: Plot of the absorbance at 260 nm vs *c***_TBAC_** (A) and at 272 nm vs *c***_SCS_** (B); straight line represents the fit of the isotherm to a 1:1 stoichiometry.

To assess whether the influence of the anions on the photoreactivity of the dibenzobarellenes is caused by complex formation, the propensity of the urea and thiourea functionalities to associate with anions was investigated by spectrophotometric titrations of selected tetrabutylammonium salts with derivatives **1h** and **1i** (Figure 1). Upon the addition of TBAC, a slight change of the absorption bands of the urea derivative **1h** was observed with the exception of the absorption maxima at 280 nm which remained essentially unaffected during the titration. The latter absorption band was assigned to the dibenzobarrelene unit, by comparison with the absorption of the resembling dibenzobarrelene derivative **1d** [45]. This observation indicates that the complexation of the chloride anion has no significant influence on the dibenzobarrelene chromophore, but rather on the trifluoromethyl-substituted phenyl substituents. The absorption of the thioureido-substituted derivative **1i** changed significantly upon the addition of the sulfonate salt **SCS**. Specifically, the absorption maximum at 272 nm was red shifted by ca. 15 nm, along with an overall increase of the absorption. In addition, an isosbestic point at 248 nm was observed during the titration process, which indicates an equilibrium between two different absorbing species, i.e., the free and complexed ligand. Because of the predominant absorption of the arylthiourea unit, it was not possible to assess the influence of complexation on the dibenzobarrelene chromophore. The binding isotherms from the spectrophotometric titration were fitted to a 1:1 stoichiometry and the resulting binding constants of the complexes were determined to be *K*_b_ = 1.1 × 10^4^ M^−1^ for **1h**-TBAC and *K*_b_ = 1.8 × 10^4^ M^−1^ for **1i**-**SCS** (Figure 1). In addition, it was observed that the ^1^H NMR spectroscopic signals of the NH protons of **1h** (from 6.63 and 9.34 to 7.66 and 10.20) as well as the one of the methine proton (from 4.54 to 4.35) and of the OH proton (from 5.22 to 5.14) of the mandelate were significantly shifted upon the addition of (*S*)-mandelate (**SMD**) [in (CD_3_)_2_SO)]. The corresponding Job plot confirms the 1:1 complex between **1h** and **SMD** (Supporting Information File 1). Moreover, complex formation was confirmed by a weak NOE effect, as determined by ROESY NMR experiments, between the protons in the ortho position of the phenyl group of the mandelate and the bis(trifluoro)phenyl groups of **1h**.

**Figure 2:**
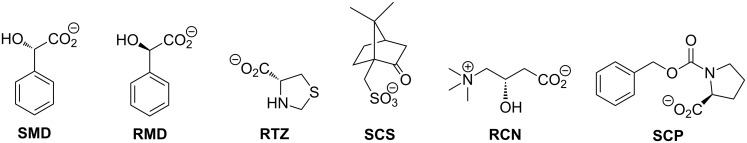
Structures of chiral additives employed in DPM rearrangements.

Since it was demonstrated that the ureido- and thioureido-substituted dibenzobarrelene derivatives **1h** and **1i** associate with anions, experiments were carried out to assess whether a stereoselective DPM rearrangement of **1h** may be induced by a bound chiral anion. The initial experiments were performed with (*S*)-mandelate (**SMD**). Thus, a complex of the dibenzobarrelene **1h** with **SMD** was irradiated in acetone solution at different concentrations and with varied host–guest ratios (Table 1, entries 1–5). The enantiomeric ratio (er) of the dibenzosemibullvalene product was determined by ^1^H NMR spectroscopy with **SMD** as chiral shift reagent, as it turned out that this additive induces a significant separation of the protons of the enantiomers of **2h** (Supporting Information File 1). The absolute configuration of the products was not determined. The photoreaction proceeded rapidly with full conversion in 4–6 hours with moderate stereoselectivity (68:32 er) in the presence of 1.1 molar equiv of the chiral mandelate. Variations of the host–guest ratio (*c***_1h_**:*c*_anion_ = 1:0.5, 1:2.1, 1:5) led to a decrease of the stereoselectivity. In addition, changes in the concentration of the dibenzobarrelene **1h** did not have a significant influence on the stereoselectivity of the reaction. Based on these results, the following experiments were carried out with a concentration of 0.25 mM for the dibenzobarrelene derivative **1h** and 1.1 molar equiv of the chiral additive (Table 1, entries 6–10). Notably, the (*R)-*enantiomer of mandelate induced the same extent of stereoselectivity with the reverse ratio of products. For comparison, the photoreaction of dibenzobarrelene **1h** was performed in the presence of other chiral anions, namely (*R*)*-*thiazolidine-4-carboxylate (**RTZ**), (*S*)-camphor-10-sulfonate (**SCS**), (*R*)-carnitine (**RCN**), and (2*S*)-1-[(benzyloxy)carbonyl]-2-pyrrolidinecarboxylate (**SCP**) (Figure 2). In each case, the induced stereoselectivity was significantly lower than that induced by (*S*)-mandelate.

**Table 1:** DPM rearrangements of dibenzobarrelene **1h** in the presence of chiral anions.

Entry	Solvent^a^	Anion^b^	*c***_1h_** / mM	*c***_1h_**:*c***_anion_**	er^c^

1	Acetone	**SMD**	0.25	1:0.5	45:55
2	Acetone	**SMD**	0.25	1:1.1	32:68
3	Acetone	**SMD**	0.50	1:1.1	33:67
4	Acetone	**SMD**	0.50	1:2.1	41:59
5	Acetone	**SMD**	0.50	1:5.0	47:53
6	Acetone	**RMD**	0.25	1:1.1	67:33
7	Acetone	**RTZ**	0.25	1:1.1	59:41
8	Acetone	**SCS**	0.25	1:1.1	44:56
9	Acetone	**SCP**	0.25	1:1.1	53:47
10	MeCN-MeOH 1:1	**RCN**	0.25	1:1.1	47:53
11	Acetonitrile	**SMD**	0.25	1:1.1	40:60
12	Methanol	**SMD**	0.25	1:1.1	48:52
13	Ethanol	**SMD**	0.25	1:1.1	49:51
14	2-Propanol	**SMD**	0.25	1:1.1	50:50
15	Acetone-THF 1:9	**SMD**	0.25	1:1.1	45:55
16	Acetone-EtOAc 1:9	**SMD**	0.25	1:1.1	35:65
17	Acetone-Benzene 1:9	**SMD**	0.25	1:1.1	44:56

^a^Conditions described in the Experimental Section; amount of **2h** in reaction mixture: >90% in all cases. ^b^Except for **SCN** used as tetrabutylammonium salts. ^c^er = enantiomeric ratio, determined by ^1^H NMR spectroscopic analysis with 5 molar equiv of **SMD** as the shift reagent; estimated error: ±3% of the given data. Each measurement was carried out twice to ensure the reproducibility.

The influence of the solvent on the DPM rearrangement of compound **1h** was also investigated in the presence of (*S*)-mandelate (Table 1, entries 11–17). The low solubility of **1h** in non-polar solvents required the addition of 10% acetone as co-solvent to give a homogeneous solution. Notably, a small but significant stereoselectivity of the DPM rearrangement of **1h** was only observed in acetone or ethyl acetate/acetone (32:68 and 35:65 er), whereas in acetonitrile (40:60 er), THF (45:55 er) or MeOH, EtOH or 2-PrOH (50:50 er) the DPM rearrangement of compound **1h** proceeds with very low or no selectivity.

In additional experiments, the photoreactions of the thioureido-substituted dibenzobarrelene derivative **1i** were studied with chiral mandelate, camphorsulfonate and binaphthyl phosphonate in a variety of solvents including acetone, acetonitrile, ethyl acetate, dichloromethane, and benzene. Although the DPM rearrangement of the dibenzobarrelene **1i** took place readily upon irradiation and the semibullvalene photoproducts were isolated by column chromatography in very good yields, none of these products proved to be enantiomerically enriched, as determined by ^1^H NMR experiments with **SMD** as the chiral shift reagent.

## Discussion

It is well established that the regio- and stereoselectivity of a photoreaction may be induced by the selective preorganization of the substrates by hydrogen bonding between neutral organic functionalities with an appropriate substitution pattern or by complexation of crown-ether units to cationic guest molecules [1–3]. In contrast, the selective anion recognition has not been employed systematically to influence the photochemical properties of a substrate, although such supramolecular receptor–anion interactions have been used in the organocatalysis of ground-state reactions [40–44]. It should be noted that the supramolecular interactions between anions and ureido- or thioureido-substituted fluorophores have been used elegantly for the fluorimetric detection of the anion [46], and the photophysical background has been evaluated in detail [47], but the influence of the binding event on the photochemical properties has not been assessed. In this regard, the studies of the photoreactivity of the dibenzobarrelene derivatives **1h** and **1i** provide useful initial information about the potential of anion-controlled photoreactions.

**Figure 3:**
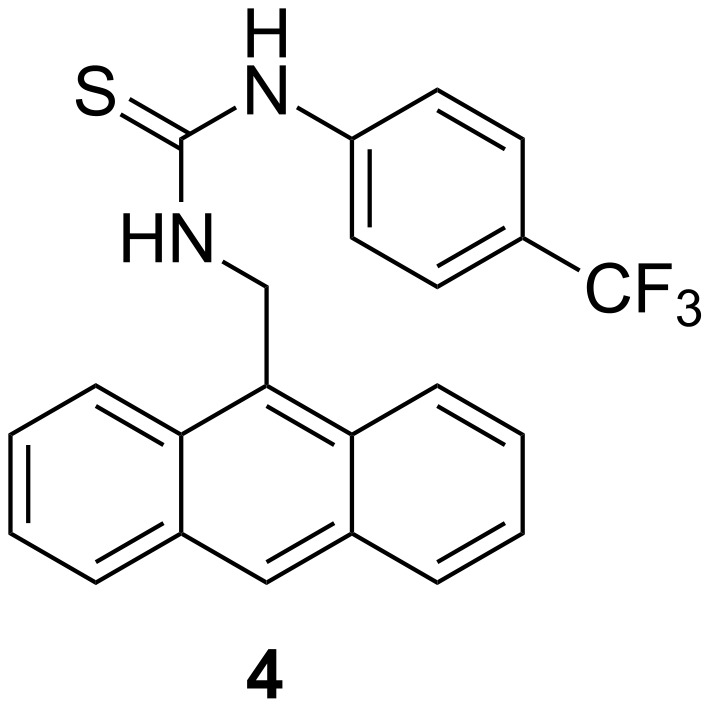
Structure of anthracene–thiourea conjugate **4**.

The fact that the DPM rearrangement of ureido-substituted dibenzobarrelene derivative **1h** takes place even without external sensitizers suggests that an efficient intersystem crossing (ISC) process exists for the excited chromophore **1h** that directs the photoreaction predominately to the triplet pathway. The 3,5-bis(trifluoromethyl)phenyl substituent may be responsible for the ISC, because *m-*bis(trifluoromethyl)benzene has an ISC quantum yield of Φ_ISC_ = 0.12 (λ_ex_ = 254 nm) in the gas phase, and the latter compound is able to sensitize a triplet-state *E/Z-*isomerization of alkenes [48]. On the other hand, the thioureido-substituted dibenzobarrelene derivative **1i** does not undergo a DPM rearrangement upon direct irradiation, despite the potentially sensitizing 3,5-bis(trifluoromethyl)phenyl substituents. Notably, not even the commonly employed sensitizer acetone is capable of inducing the DPM rearrangement of **1i**. Considering the different photophysical and photochemical properties of the carbonyl and thiocarbonyl chromophores [49], it may be that a similar difference exists between urea and thiourea functionalities. Thiocarbonyl groups usually have high ISC rates, but they are also prone to self-quenching [49] and act as efficient quenchers for triplet reactions [50]. Thus, in analogy to the properties of the thiocarbonyl chromophore, it is proposed that the thioureido functionality in **1i** quenches the triplet excited-state, most likely by the intramolecular self-quenching of the two proximate thiourea groups. Interestingly, upon association of the thiourea units with anions, the DPM reactivity of compound **1i** is regained. This observation is consistent with the shorter reaction time of the DPM rearrangement of the ureido-substituted derivative **1h** upon association with anions. Since the photometric titrations clearly indicate complex formation, it may be assumed that the decreased reaction times are due to restricted molecular flexibility of the ureido- and thioureido substituents within the complex. Specifically, the deactivation of the excited-state by conformational relaxation is suppressed upon complex formation leading to increased quantum yields of the photoreaction. Nevertheless, in the case of the thioureido-substituted derivative **1i** additional effects need to be considered to explain the drastic change of the photochemical properties. Apparently, the quenching effect of the thioureido substituents on the triplet reaction is no longer effective after the association with anions. Presumably, the complexed anions affect the properties of the C=S bond in **1i**, leading to changes in excited-state reactivity, as has been shown for hydrogen bonded thiocarbonyl compounds in a theoretical study [51]. For comparison, it should be noted that the ISC rate constant of the thioureido-substituted anthracene **4** (Figure 3), *k*_ISC_ = 1.1 × 10^9^ s^−1^ (CH_3_CN), even decreases by one order of magnitude upon association with acetate [27]. In that case, however, the absorption of the anthracene and the (trifluoromethyl)phenylthiourea part are well separated and the anthracene is excited selectively at lower energy. Moreover, as there is only one thioureido substituent attached to the anthracene in **4**, self-quenching can only take place in a bimolecular process, which is negligible at the low concentration employed in these experiments.

**Figure 4:**
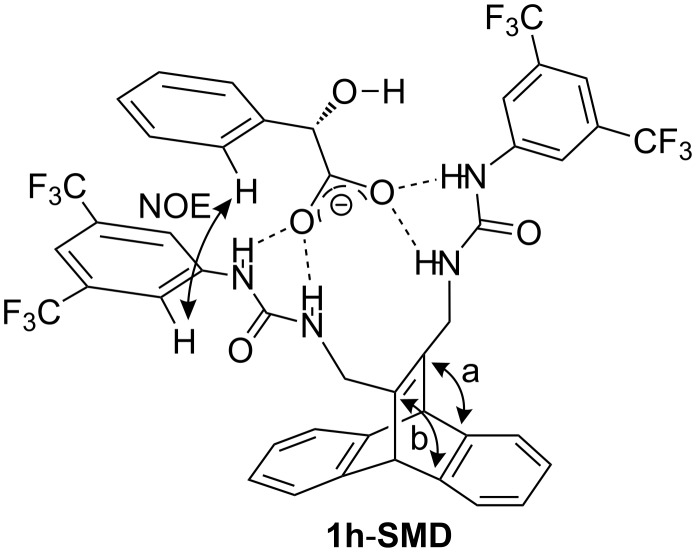
Proposed structure of the complex between **1h** and mandelate **SMD**.

It was demonstrated that the complexation of chiral carboxylates by the ureido substituents of the dibenzobarrelene derivative **1h** may be employed, in principle, to induce a stereoselective DPM rearrangement. The lack of stereoselectivity in competitive protic solvents, namely alcohols, indicates the relevance of the hydrogen bonding between the anion and the urea group for chiral induction. As the best selectivities were observed in the presence of 1 molar equiv of the mandelate ion, it may be deduced that the stereoselectivity of the reaction mainly originates from a 1:1 complex between **1h** and the mandelate **SMD** (**1h**-**SMD**) (Figure 4), thus resembling known complexes, in which a bisurea receptor uses all four NH hydrogen for chelating hydrogen bonding to carboxylate in a 1:1 complex [52–54]. The fact that mandelate induces a significantly higher selectivity than the other employed anions may be explained by additional interactions of the hydroxy or phenyl substituent of the mandelate with the bis(trifluoromethyl)phenyl substituent or with the ureido substituent. Presumably, in complex **1h**-**SMD** one initial vinyl–benzo bridging (pathway a or b) is preferred due to steric or conformational restraints; however, this effect is only small, but significant, as indicated by the moderate stereoselectivity (68:32 er).

At present, the reason for the lack of stereoselectivity of the DPM rearrangement of the thioureido-substituted dibenzobarrelene **1i** upon complexation of chiral anions is not clear. Nevertheless, it has been shown that neighboring aryl substituents decrease the anion-binding ability of thiourea derivatives because of the steric repulsion between the ortho-substituents and the sulfur atom [55]. This effect may also suppress the formation of a stable 1:1 complex between the chelating thioureido functionalities in **1i** and anions, such that an inducing effect of the anion on the photoreaction of the dibenzobarrelene is not operative.

## Conclusion

In summary, it was demonstrated that the photochemical properties of the bisureido- and bisthioureido-substituted dibenzobarralene derivatives **1h** and **1i** may be influenced by complex formation with appropriate anions. In general, the photoreactivity of the substrates is significantly increased upon association with anions. Specifically, the DPM rearrangement of the thioureido derivative **1i** to give the dibenzosemibullvalene **2i** can only be performed successfully when the self-quenching of the triplet state is suppressed by complex formation. At the same time it was shown in preliminary experiments that the association of chiral carboxylates with **1h** induces a stereoselective DPM rearrangement. So far, the selectivities are very low; however, these observations demonstrate that anion-controlled stereoselective DPM rearrangements may be accomplished in principle. Therefore, it is proposed that this methodology may be optimized in future studies, thus providing a complementary tool to perform stereoselective photoreactions based on supramolecular interactions.

## Experimental

**General remarks:** The NMR spectra were recorded on a Bruker Avance 400 (^1^H NMR: 400 MHz; ^13^C NMR: 100 MHz) and a Varian NMR system 600 (^1^H NMR: 600 MHz; ^13^C NMR: 150 MHz). ^1^H NMR chemical shifts are relative to tetramethylsilane (δ_TMS_ = 0.00 ppm), and ^13^C NMR chemical shifts refer to either the signal of tetramethylsilane (δ_TMS_ = 0.00 ppm) or the solvent signals [(CD_3_)_2_CO: 29.8 ppm, (CD_3_)_2_SO: 39.5 ppm]. Absorption spectra were recorded on a Varian 100 Bio spectrometer at 25 °C. Melting points were determined on a Büchi 510K and are uncorrected. Mass spectra were recorded on a Hewlett-Packard HP 5968 (EI) and a Finnigan LCQ Deca instrument (ESI). Elemental analyses were performed on a KEKA-tech EuroEA combustion analyzer by Mr. H. Bodenstedt, Organic Chemistry I, University of Siegen. TLC analyses were performed on silica-gel sheets (Macherey-Nagel Polygram Sil G/UV_254_). Unless otherwise mentioned, commercially available chemicals were reagent grade and were used without further purification. Tetrabutylammonium hydroxide in MeOH (1.0 M) and (*S*)-camphor-10-sulfonic acid were obtained from Aldrich. (*R*)-Mandelic acid and (*S*)-mandelic acid were obtained from Fluka. (*R*)*-*Thiazolidine-4-carboxylic acid and (2*S*)-1-[(benzyloxy)carbonyl]-2-pyrrolidinecarboxylic acid were obtained from Acros. (*R*)-Carnitine was obtained from Alfa-Aesar. Preparative column chromatography was performed on MN Silica Gel 60 M (particle size 0.04–0.063 mm, 230–440 mesh).

Irradiations were performed with a TQ150 middle-pressure mercury lamp (Heraeus, UV-Consulting Peschl), which was placed inside a quartz cooling tube. The reaction mixture was placed ca. 10–15 cm in front of the lamp.

**General procedure for the preparation of bisurea- and bisthiourea derivatives of dibenzobarrelene (GP1):** The isocyanate or isothiocyanate derivative (1.1 molar equiv) was added to a stirred solution of 11,12-bis(aminomethyl)-9,10-dihydro-9,10-ethenoanthracene (**1d**, 0.45–10.0 mmol) [45] in CH_2_Cl_2_ (3 mL/mmol) at 0 °C. A white or pale yellow solid precipitated shortly after the addition of iso(thio)cyanate. The mixture was stirred for 2 h at room temperature, and the solid collected by filtration or recrystallization directly from the reaction mixture depending on the solubility of the product.

**11,12-Bis(*N*’-*n*-butylureidomethyl)-9,10-dihydro-9,10-ethenoanthracene** (**1e**)**:** Prepared from dibenzobarrelene **1d** (554 mg, 2.00 mmol) according to **GP1**, collected by filtration and dried in vacuo. Yield 732 mg (1.77 mmol, 79%), white powder, mp > 320 °C (dec.). ^1^H NMR [400 MHz, (CD_3_)_2_SO]: δ 0.88 (t, *J* = 7 Hz, 6H, CH_3_), 1.23–1.34 (m, 8H, CH_2_), 2.95–2.99 (m, 4H, C*H*_2_NH), 3.83 (d, *J* = 7 Hz, 4H, C*H*_2_C=C), 5.08 (s, 2H, CH), 5.83–5.85 (m, 4H, NH), 6.91, 7.22 (AA’BB’-system, 8H, CH_ar_). ^13^C NMR [100 MHz, (CD_3_)_2_SO]: δ 14.1 (CH_3_), 19.9 (CH_2_), 32.6 (CH_2_), 38.0 (CH_2_), 53.1 (CH), 122.9 (CH_ar_), 124.5 (CH_ar_), 143.3 (C_q_), 146.6 (C_q_), 158.6 (C=O). Anal. Calcd for C_34_H_28_N_4_O_2_ (460.6): C, 73.01; H, 7.88; N, 12.16. Found: C, 73.25; H, 7.95; N, 12.04.

**11,12-Bis(*N*’-phenylureidomethyl)-9,10-dihydro-9,10-ethenoanthracene** (**1f**): Prepared from dibenzobarrelene **1d** (131 mg, 0.50 mmol) according to **GP1** and collected by filtration and obtained as a white powder (179 mg, 0.36 mmol, 72%), mp 309–312 °C (dec.). ^1^H NMR [400 MHz, (CD_3_)_2_SO]: δ 4.07 (d, *J* = 5 Hz, 4H, CH_2_), 5.15 (s, 2H, CH), 6.37 (t, *J* = 5 Hz, 2H, NH), 6.90–6.93 (m, 6H, CH_ar_), 7.20–7.41 (m, 12H, CH_ar_), 8.29 (s, 2H, NH). ^13^C NMR [100 MHz, (CD_3_)_2_SO]: δ 37.9 (CH_2_), 53.3 (CH), 118.3 (CH_ar_), 121.5 (CH_ar_), 123.0 (CH_ar_), 124.6 (CH_ar_), 129.0 (CH_ar_), 140.8 (C_q_), 143.5 (C_q_), 146.5 (C_q_), 155.8 (C=O). MS (EI): *m*/*z* = 500 [M^+^]. Anal. Calcd for C_34_H_28_N_4_O_2_ (500.6): C, 76.78; H, 5.64; N, 11.19. Found: C, 76.68; H, 5.67; N, 11.12.

**11,12-Bis[*N*’-(4-*n*-butylphenyl)ureidomethyl]-9,10-dihydro-9,10-ethenoanthracene** (**1g**): Prepared from dibenzobarrelene **1d** (0.12 g, 0.45 mmol) according to **GP1**, collected by filtration and dried in vacuo. White amorphous solid, yield 0.21 g (0.34 mmol, 76%), mp > 320 °C. ^1^H NMR [400 MHz, (CD_3_)_2_SO]: δ 0.89 (t, *J* = 7 Hz, 6H, CH_3_), 1.26–1.55 (m, 8H, C*H_2_*C*H_2_*CH_3_), 2.47–2.50 (m, 4H, PhC*H_2_*CH_2_, partly overlapped with the solvent signal), 3.97 (d, *J* = 6 Hz, 4H, C=CC*H_2_*), 5.14 (s, 2H, CH), 6.06 (t, *J* = 6 Hz, 2H, NH), 6.90–6.92 (m, 4H, CH_ar_), 7.03–7.05 (m, 4H, CH_ar_), 7.25–7.29 (m, 8H, CH_ar_), 8.42 (s, 2H, NH). ^13^C NMR [100 MHz, (CD_3_)_2_SO]: δ 14.2 (CH_3_), 22.1 (CH_2_), 33.7 (CH_2_), 34.5 (CH_2_), 37.9 (CH_2_), 53.3 (CH), 118.4 (CH_ar_), 123.0 (CH_ar_), 124.6 (CH_ar_), 128.8 (CH_ar_), 135.3 (CH_ar_), 138.4 (C_q_), 143.5 (C_q_), 146.5 (C_q_), 155.9 (C=O). MS (EI): *m*/*z* (%) = 613 [M^+^]. Anal. Calcd for C_40_H_44_N_4_O_2_ (612.8): C, 78.40; H, 7.24; N, 9.14. Found: C, 78.12; H, 7.25; N, 9.09.

**11,12-Bis{*N*’-[3,5-bis(trifluoromethyl)phenyl]ureidomethyl}-9,10-dihydro-9,10-ethenoanthracene** (**1h**)**:** Prepared from dibenzobarrelene **1d** (0.13 g, 0.50 mmol) according to **GP1**. After filtration of the precipitate, the product was purified by recrystallization from CH_2_Cl_2_/hexane and obtained as a white solid (0.33 g, 0.41 mmol, 82%), mp > 300 °C. ^1^H NMR [400 MHz, (CD_3_)_2_CO]: δ 4.18 (d, *J =* 4 Hz, 4H, CH_2_), 5.25 (s, 2H, CH), 6.43 (br s, 2H, NH), 6.86 (m, 4H, CH_ar_), 7.27 (m, 4H, CH_ar_), 7.51 (s, 2H, CH_ar_), 8.03 (br s, 4H, CH_ar_), 8.64 (br s, 2H, NH). ^13^C NMR [100 MHz, (CD_3_)_2_CO]: δ 38.3 (CH_2_), 53.3 (CH), 113.9 (CH_ar_), 117.7 (CH_ar_), 122.4 (CH_ar_), 123.0 (CH_ar_), 124.6 (CH_ar_), 125.1 (CH_ar_), 130.7 (CH_ar_), 131.1 (CH_ar_), 142.7 (C_q_), 143.4 (C_q_), 146.4 (C_q_), 155.4 (C=O). UV (MeCN): λ_max_ (log ε) = 229 (4.49), 246 (4.86), 272 (4.01), 280 (4.13). MS (ESI^–^): *m*/*z* (%) = 771 (100) [M − H]^−^. Anal. Calcd for C_36_H_24_F_12_N_4_O_2_ (772.6): C, 55.97; H, 3.13; N, 7.25. Found: C, 55.58; H, 2.85; N, 7.04.

**11,12-Bis*{N*’-[3,5-(bistrifluoromethyl)phenyl]thioureidomethyl}-9,10-dihydro-9,10-ethenoanthracene** (**1i**)**:** Prepared from dibenzobarrelene **1d** (0.13 g, 0.50 mmol) according to **GP1** and obtained by recrystallization from CH_2_Cl_2_/hexane as a white solid (0.34 g, 0.43 mmol, 86%), mp > 300 °C. ^1^H NMR [600 MHz, (CD_3_)_2_CO]: δ 4.66 (d, *J =* 4 Hz, 4H, CH_2_), 5.36 (s, 2H, CH), 6.92 (m, 4H, CH_ar_), 7.30–7.32 (m, 4H, CH_ar_), 7.73 (m, 4H, CH_ar_ overlapped with NH), 8.25–8.26 (m, 4H, CH_ar_), 9.57 (br s, 2H, NH). ^13^C NMR [150 MHz, (CD_3_)_2_CO]: δ = 43.4 (CH_2_), 54.2 (CH), 117.9 (CH_ar_), 121.5 (CH_ar_), 123.6 (CH_ar_), 125.3 (CH_ar_), 127.0 (CH_ar_), 132.0 (CH_ar_), 142.4 (C_q_), 144.1 (C_q_), 146.8 (C_q_), 182.4 (C=O). UV (MeCN): λ_max_ (log ε) = 230 (4.49), 246 (4.59), 272 (4.46), 280 (4.47). MS (ESI): *m*/*z* (%) = 803 (100) [M − H]^−^. Anal. Calcd for C_36_H_24_F_12_N_4_S_2_ (804.7): C, 53.73; H, 3.01; N, 6.96; S, 7.97. Found: C, 53.58; H, 2.79; N, 6.84; S, 7.97.

**General procedure for the synthetic photolysis in solution (GP2):** Solutions of the substrate (10^−3^–10^−2^ mol/l) were placed in a Duran flask (acetone) or quartz test tube (other solvents), and argon gas was bubbled carefully through the solution for at least 20 min. The solution was irradiated for 4–15 h with stirring until the starting material was fully converted as determined by TLC or ^1^H NMR spectroscopic analysis. After evaporation of the solvent in vacuo, the photolysate was analyzed by ^1^H NMR spectroscopy. In preparative experiments, the photoproduct was isolated by recrystallization or column chromatography.

**4b,8b-Dihydro-8c,8e-bis{*N*’-[3,5-bis(trifluoromethyl)phenyl]ureidomethyl}dibenzo[*a*,*f*]cyclopropa[*c*,*d*]pentalene** (**2h**)**:** Prepared by irradiation of **1h** (48.0 mg, 0.06 mmol) in acetone solution according to **GP2** and obtained as white crystals (29.0 mg, 0.04 mmol, 60%), mp 246–247 °C. ^1^H NMR [600 MHz, (CD_3_)_2_CO]: δ 3.22 (s, 1H, CH), 3.78 (dd, *J =* 15, 6 Hz, 1H, CH_2_), 3.82 (dd, *J =* 15, 6 Hz, 1H, CH_2_), 3.94 (dd, *J =* 15, 6 Hz, 1H, CH_2_), 4.44 (dd, *J =* 15, 6 Hz, 1H, CH_2_), 4.63 (s, 1H, CH), 6.42 (t, *J* = 5 Hz, 1H, NH), 6.53 (t, *J* = 5 Hz, 1H, NH), 6.99–7.05 (m, 4H, CH_ar_), 7.21–7.25 (m, 3H, CH_ar_), 7.36–7.38 (m, 1H, CH_ar_), 7.49 (s, 2H, CH_ar_), 8.09 (s, 2H, CH_ar_), 8.13 (s, 2H, CH_ar_), 8.75 (s, 1H, NH), 8.81 (s, 1H, NH). ^13^C NMR [150 MHz, (CD_3_)_2_CO]: δ 41.3 (CH_2_), 41.4 (CH_2_), 46.7 (CH), 53.3 (C_q_), 58.8 (C_q_), 67.4 (CH), 115.6 (CH_ar_), 115.6 (CH_ar_), 115.6 (CH_ar_), 115.7 (CH_ar_), 119.3 (CH_ar_), 119.3 (CH_ar_), 123.4 (CH_ar_), 123.4 (CH_ar_), 124.5 (CH_ar_), 124.6 (C_q_), 125.8 (CH_ar_), 126.3 (C_q_), 126.4 (CH_ar_), 126.6 (CH_ar_), 128.1 (CH_ar_), 128.2 (CH_ar_), 128.3 (CH_ar_), 128.5 (CH_ar_), 133.2 (C_q_), 133.2 (C_q_), 133.4 (C_q_), 133.4 (C_q_), 139.9 (CH_ar_), 140.1 (CH_ar_), 144.4 (C_q_), 144.6 (C_q_), 151.8 (C_q_), 152.8 (C_q_), 156.7 (C=O), 157.2 (C=O). MS (ESI): *m*/*z* (%) = 771 (100) [M − H]^−^. An analytical sample was obtained by recrystallization from ethyl acetate/hexane and contained one equiv of ethyl acetate as the lattice solvent. Anal. Calcd for C_36_H_24_F_12_N_4_O_2_·EtOAc (860.7): C, 55.82; H, 3.75; N, 6.51. Found: C, 55.93; H, 3.51; N, 6.45.

**4b,8b-Dihydro-8c,8e-bis{*N*’-[3,5-bis(trifluoromethyl)phenyl]thioureidomethyl}dibenzo[*a*,*f*]cyclopropa[*c*,*d*]pentalene** (**2i**)**:** Prepared by photoreaction of **1i** (0.20 g, 0.25 mmol) in acetonitrile solution in the presence of 2 molar equiv of ammonium chloride according to **GP2**. After the irradiation the inorganic components were removed by column filtration (SiO_2_; EtOAc/hexane 1/2, v/v), and the residue was recrystallized from ethyl acetate/hexane to give light yellow crystals (104 mg, 0.13 mmol, 52%), mp 181–182 °C. ^1^H NMR [600 MHz, (CD_3_)_2_CO]: δ 1.20 (t, *J =* 7 Hz, 3H, CH_3_), 3.43 (s, 1H, CH), 4.05 (q, *J =* 7 Hz, 2H, CH_2_), 4.22 (d, *J =* 13 Hz, 1H, NHC*H*HC), 4.29 (d, *J =* 13 Hz, 1H, NHC*H*HC), 4.48 (d, *J =* 13 Hz, 1H, NHCH*H*C), 4.74 (d, *J =* 13 Hz, 1H, NHCH*H*C), 4.76 (s, 1H, CH), 7.00–7.09 (m, 5H, CH_ar_), 7.24–7.28 (m, 3H, CH_ar_), 7.36–7.38 (m, 1H, CH_ar_), 7.67 (s, 2H, CH_ar_), 7.81 (s, 1H, NH), 7.89 (s, 1H, NH), 8.23 (s, 2H, CH_ar_), 8.32 (s, 2H, CH_ar_), 9.45 (s, 1H, NH), 9.53 (s, 1H, NH). ^13^C NMR [150 MHz, (CD_3_)_2_CO)]: δ 14.5 (CH_3_), 20.8 (CH_2_), 44.2 (CH_2_), 45.2 (CH_2_), 50.3 (C_q_), 57.2 (CH), 59.7 (CH), 65.1 (C_q_), 116.5 (CH_ar_), 116.7 (c), 121.5 (CH_ar_), 121.6 (CH_ar_), 122.1 (CH_ar_), 122.5 (CH_ar_), 123.8 (CH_ar_), 124.3 (CH_ar_), 124.3 (CH_ar_), 124.9 (CH_ar_), 126.4 (CH_ar_), 126.4 (CH_ar_), 126.6 (CH_ar_), 126.9 (CH_ar_), 130.6 (CH_ar_), 130.6 (CH_ar_), 130.8 (CH_ar_), 130.9 (CH_ar_), 131.1 (C_q_), 131.1 (C_q_), 137.6 (C_q_), 137.7 (C_q_), 141.8 (C_q_), 142.0 (C_q_), 149.9 (C_q_), 150.8 (C_q_), 181.4 (C=S), 181.8 (C=S), two C_q_ signals are overlapped in the aromatic region. MS (ESI): *m*/*z* (%) = 803 (100) [M − H]^−^. Anal. Calcd for C_36_H_24_F_12_N_4_S_2_·EtOAc (892.8): C, 53.81; H, 3.61; N, 6.28; S, 7.18. Found: C, 54.03; H, 3.28; N, 6.32; S, 7.09.

**Preparation of the tetrabutylammonium salts of chiral acids:** The tetrabutylammonium salts of the chiral carboxylates **SMD**, **RMD**, **RTZ**, and **SCP** were prepared by the neutralization of the corresponding chiral acids with tetrabutylammonium hydroxide (1.0 M in MeOH) [56]. The resulting salts were used as 0.1 M stock solutions in acetone.

The tetrabutylammonium salt of (S)-camphor-10-sulfonate **SCS** was prepared according to the literature procedure [57], and used as 0.1 M stock solution in the respective solvent required for the experiment.

**Photoreaction of dibenzobarrelene derivatives 1h and 1i in the presence of chiral anions:** The dibenzobarrelene derivatives **1h** or **1i** (50 μmol) were dissolved in a stock solution (0.55 mL of 0.1 M stock solution, 55 μmol, 1.1 equiv) of the chiral tetrabutylammonium salt, and the solvent was removed in vacuo. The resulting residue was re-dissolved in the solvent of choice (200 mL). Argon gas was bubbled through the solution for 20 min to remove residual oxygen from the solvent. The reaction container (DURAN, λ > 310 nm) was placed ca. 15 cm in front of the light source, and the solution was irradiated for 3–4 h (TLC control). The solvent was removed in vacuo and the major photoproduct purified by column chromatography (SiO_2_; hexane:acetone:ethyl acetate = 4:1:1, v/v/v). The photoproduct was analyzed by ^1^H NMR spectroscopy in the presence of 5 equiv of tetrabutylammonium **SMD** as chiral shift reagent. The enantiomeric ratio of the semibullvalene mixture was determined by the integration of the aromatic proton signals from each isomer (δ in the range of 8.4–8.7 ppm), and by the integration of the NH and cyclopropane CH signals. Each spectroscopic measurement was repeated twice to ensure the reproducibility.

## Supporting Information

^1^H NMR and ^13^C NMR spectra of compounds **1e**–**i** and **2h**–**i**; ^1^H NMR spectra of **2h** with (*S*)-mandelate (**SMD**) at different molar ratios and corresponding Job plot; ^1^H NMR spectra of **2h** with (*S*)-mandelate (**SMD**) as chiral shift reagent.

File 1 Supporting Information for: Effects of anion complexation on the photoreactivity of bisureido- and bisthioureido-substituted dibenzobarrelene derivatives.
