# Supplementary material for: Effects of anion complexation on the photoreactivity of bisureido- and bisthioureido-substituted dibenzobarrelene derivatives
Source: Beilstein J Org Chem. 2011 Mar 4;7:278–89. doi: 10.3762/bjoc.7.37 (PMC3063053; doi:10.3762/bjoc.7.37)
Supplement: File 1 — Supporting Information for: Effects of anion complexation on the photoreactivity of bisureido- and bisthioureido-substituted dibenzobarrelene derivatives. [file Beilstein_J_Org_Chem-07-278-s001.pdf]

Supporting Information

for

Effects of anion complexation on the photoreactivity of bisureido- and bisthioureido-substituted dibenzobarrelene derivatives

Heiko Ihmels* and Jia Luo

Address: Organic Chemistry II, University of Siegen, Adolf-Reichwein-Str. 2, D-57068 Siegen, Germany

Email: Heiko Ihmels* - ihmels@chemie.uni-siegen.de

* Corresponding author

^{1}H NMR and ^{13}C NMR Spectra of Dibenzobarrelene and Semibullvalene Derivatives

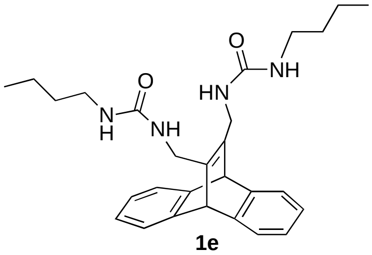

1e in $(CD_3)_2SO$

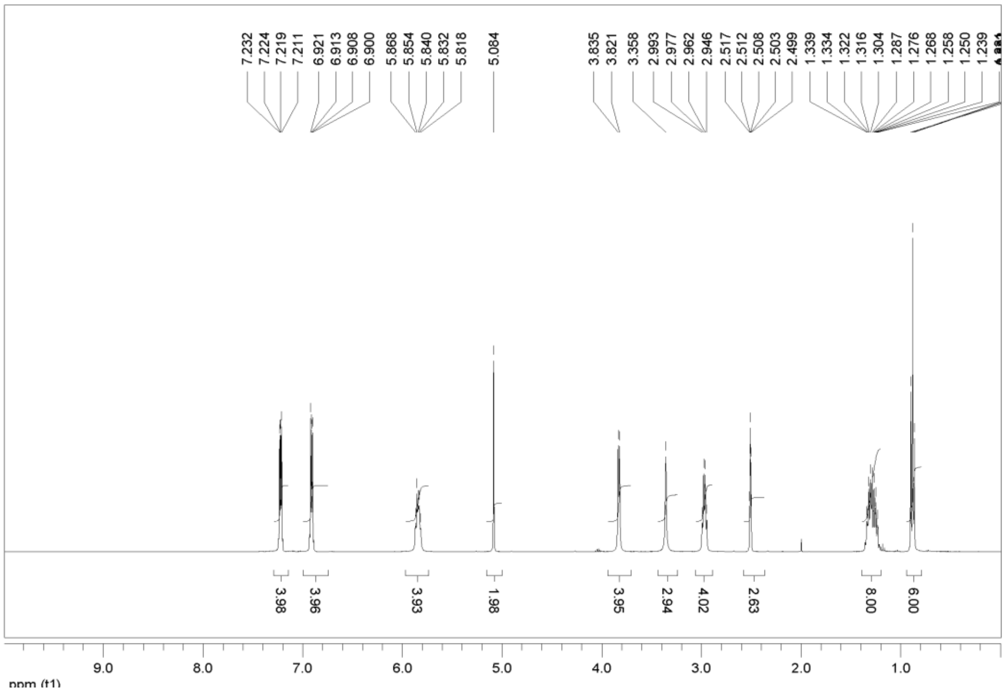

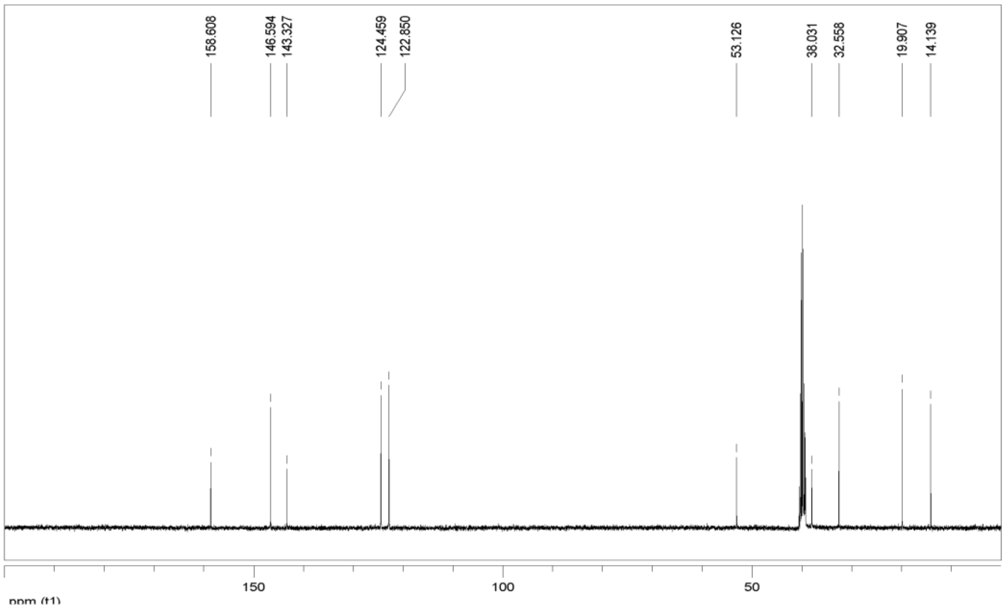

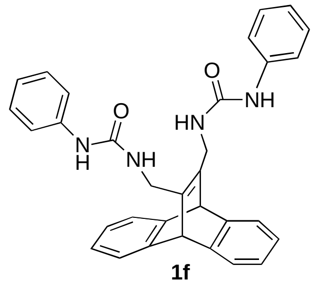
O
NH
HN
O
N
H
NH

1f

(in $(CD_3)_2SO$)

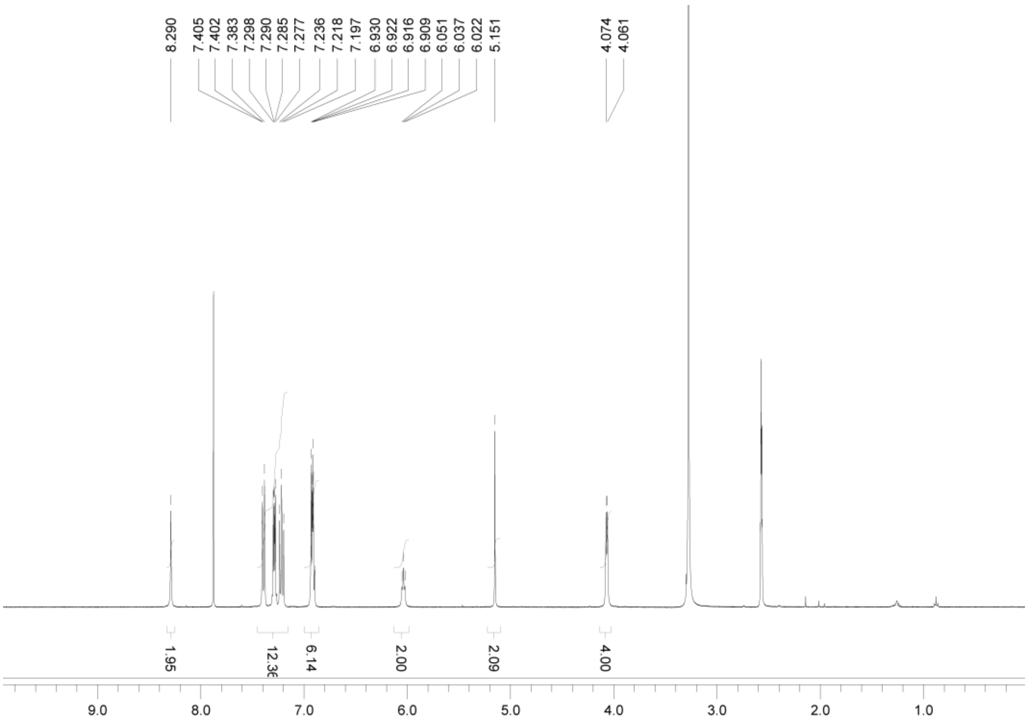
8.290
7.405
7.402
7.383
7.298
7.290
7.285
7.277
7.236
7.218
7.197
6.930
6.922
6.916
6.909
6.051
6.037
6.022
5.151
4.074
4.061
1.95
12.36
6.14
2.00
2.09
4.00
9.0
8.0
7.0
6.0
5.0
4.0
3.0
2.0
1.0

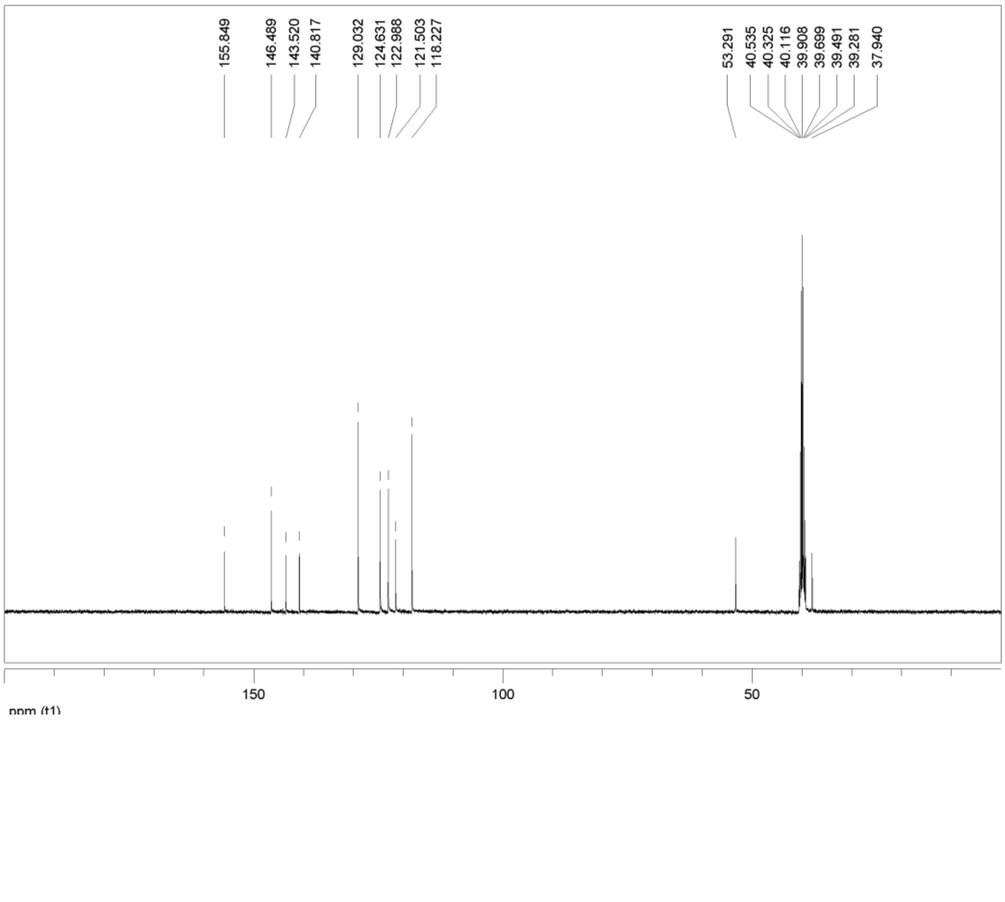
155.849
146.489
143.520
140.817
129.032
124.631
122.988
121.503
118.227
53.291
40.535
40.325
40.116
39.908
39.699
39.491
39.281
37.940
150
100
50
ppm (t1)

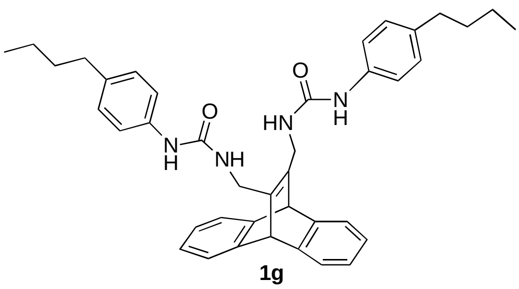
O
NH
H
NH
O
HN
N
H

1g

in $(CD_3)_2SO$

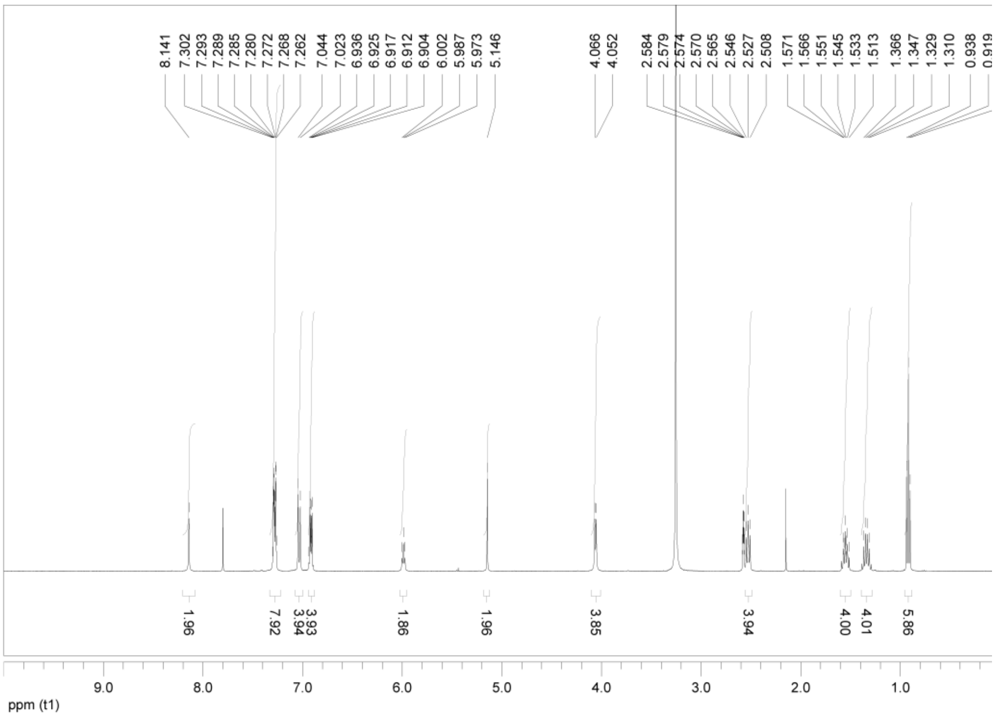
8.141
7.302
7.293
7.289
7.285
7.280
7.272
7.268
7.262
7.044
7.023
6.936
6.925
6.917
6.912
6.904
6.002
5.987
5.973
5.146
4.066
4.052
2.584
2.579
2.574
2.570
2.565
2.546
2.527
2.508
1.571
1.566
1.551
1.545
1.533
1.513
1.366
1.347
1.329
1.310
0.938
0.919
1.96
7.92
3.94
3.93
1.86
1.96
3.85
3.94
4.00
4.01
5.86
9.0
8.0
7.0
6.0
5.0
4.0
3.0
2.0
1.0
ppm (t1)

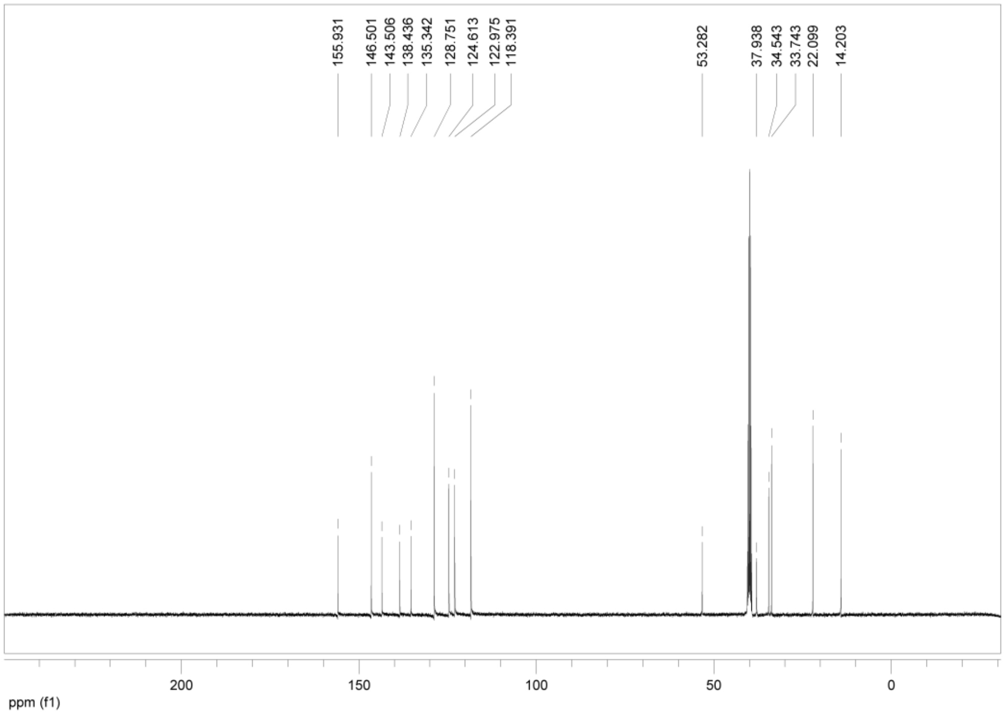
155.931
146.501
143.506
138.436
135.342
128.751
124.613
122.975
118.391
53.282
37.938
34.543
33.743
22.099
14.203
200
150
100
50
0
ppm (f1)

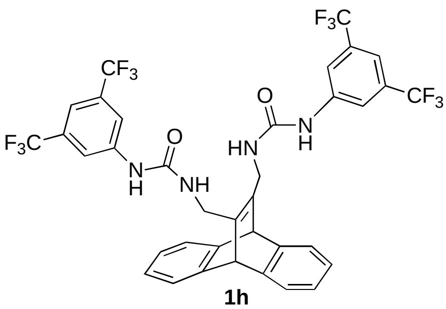
CF3
F3C
O
N
H
NH
O
HN
N
H
F3C
CF3

1h

in $(CD_3)_2CO$

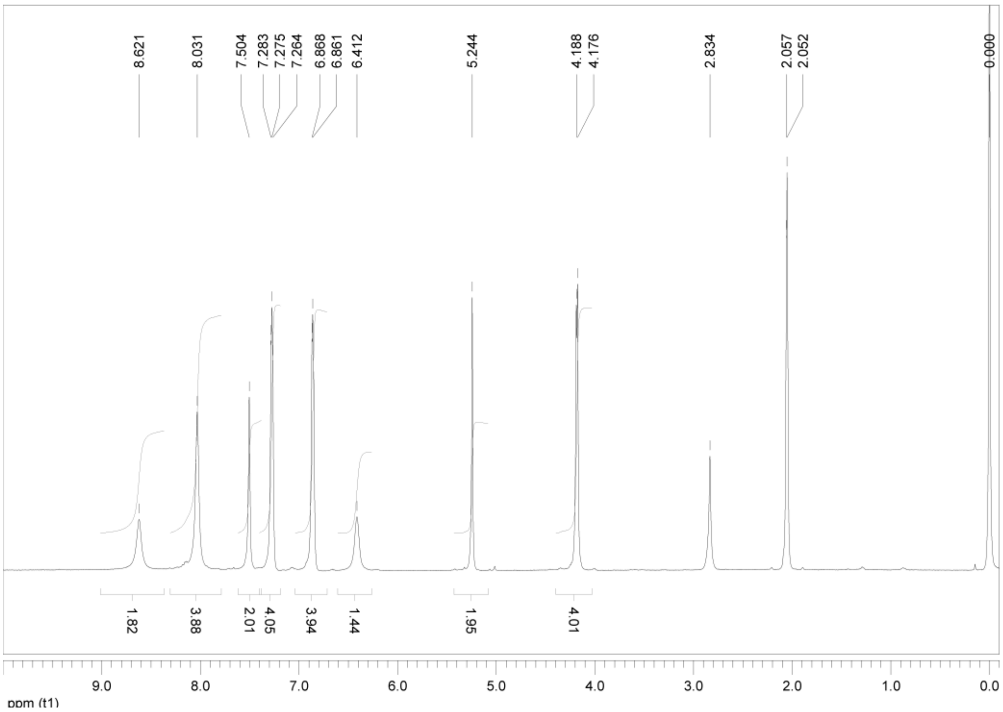
8.621
8.031
7.504
7.283
7.275
7.264
6.868
6.861
6.412
5.244
4.188
4.176
2.834
2.057
2.052
0.000
1.82
3.88
2.01
4.05
3.94
1.44
1.95
4.01
9.0
8.0
7.0
6.0
5.0
4.0
3.0
2.0
1.0
0.0
ppm (t1)

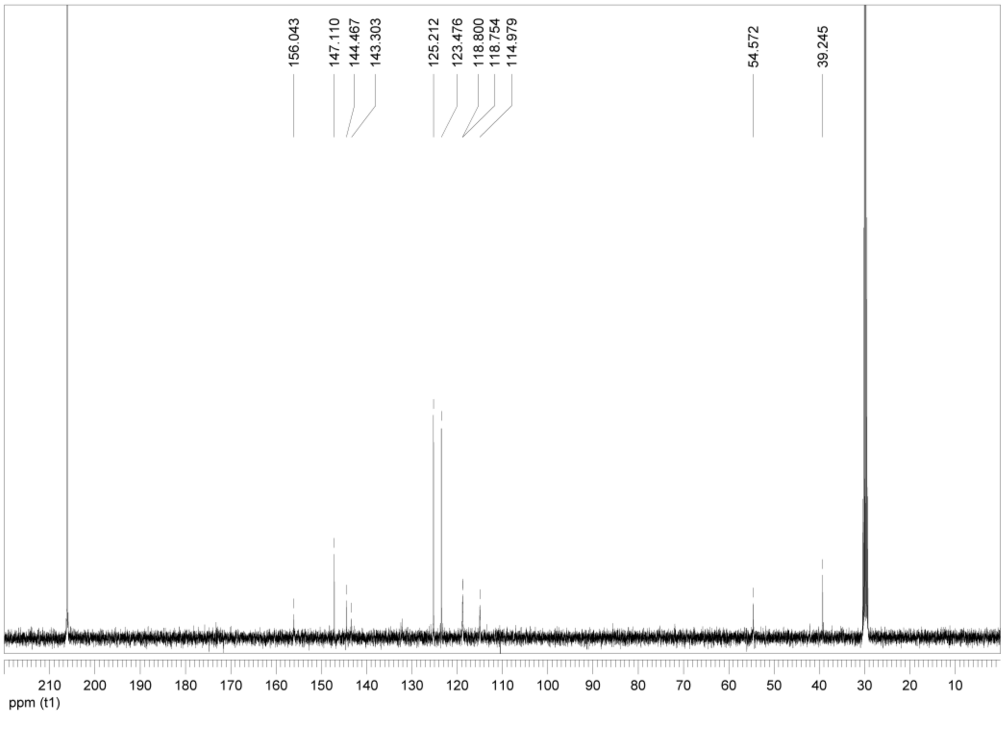
156.043
147.110
144.467
143.303
125.212
123.476
118.800
118.754
114.979
54.572
39.245
210
200
190
180
170
160
150
140
130
120
110
100
90
80
70
60
50
40
30
20
10
ppm (t1)

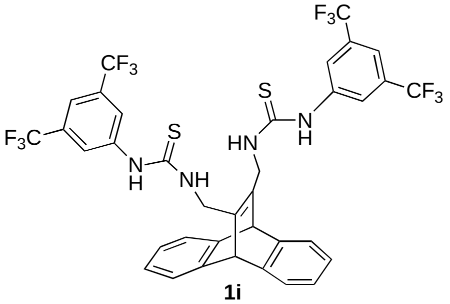

F_3C
CF_3
F_3C
CF_3
S
S
N
H
NH
HN
N
H

1i

in $(CD_3)_2CO$

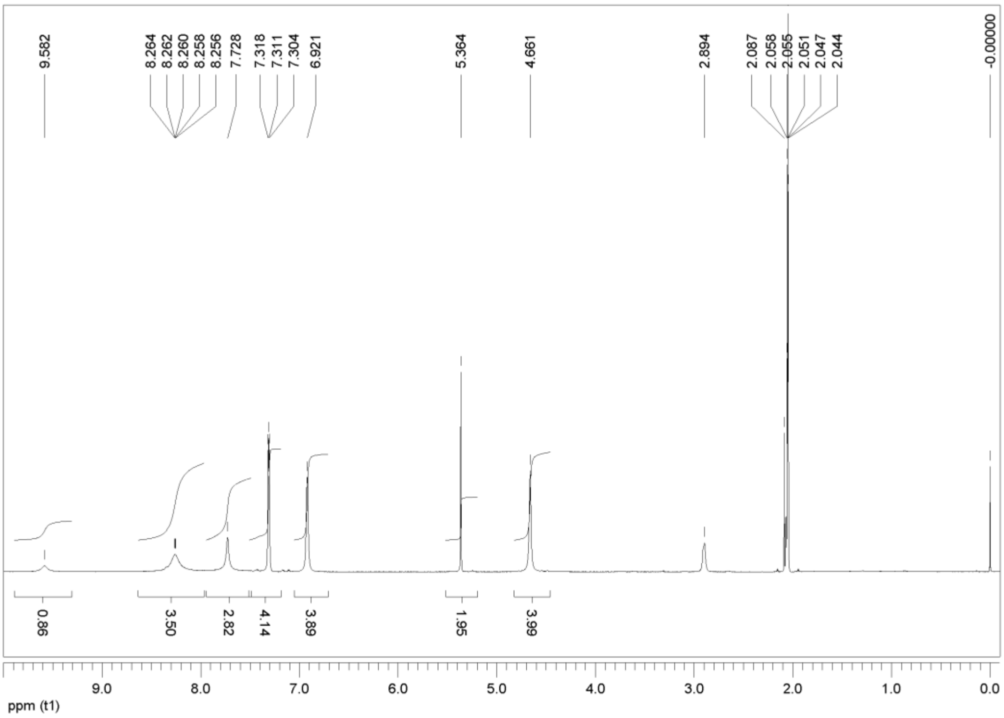

9.582
8.264
8.262
8.260
8.258
8.256
7.728
7.318
7.311
7.304
6.921
5.364
4.661
2.894
2.087
2.058
2.055
2.051
2.047
2.044
-0.00000
0.86
3.50
2.82
4.14
3.89
1.95
3.99
9.0
8.0
7.0
6.0
5.0
4.0
3.0
2.0
1.0
0.0
ppm (t1)

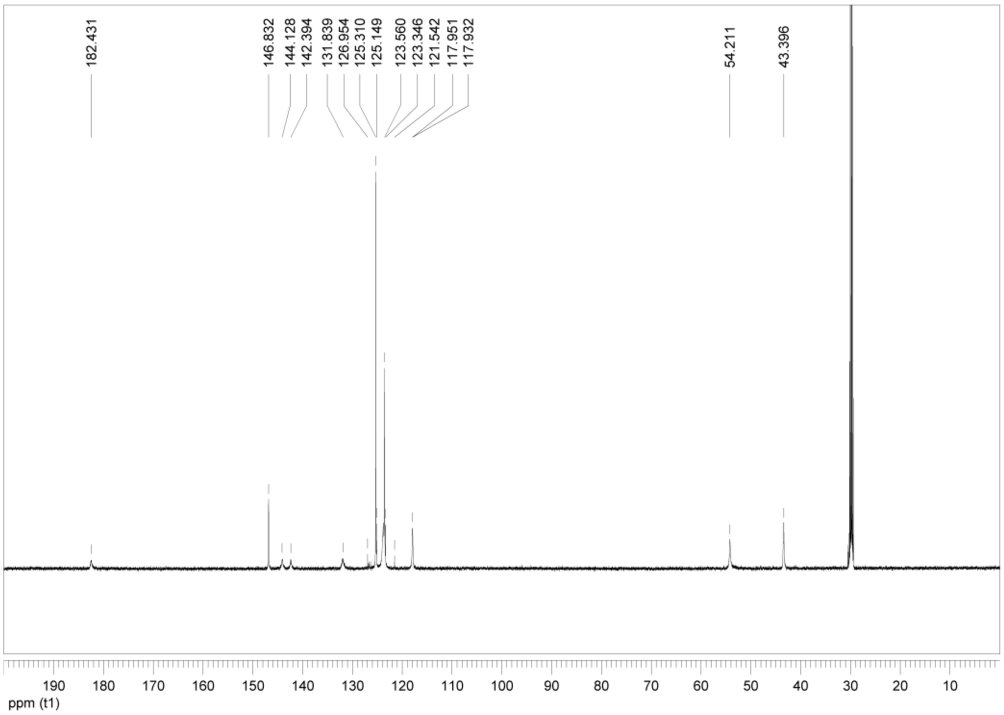

182.431
146.832
144.128
142.394
131.839
126.954
125.310
125.149
123.560
123.346
121.542
117.951
117.932
54.211
43.396
190
180
170
160
150
140
130
120
110
100
90
80
70
60
50
40
30
20
10
ppm (t1)

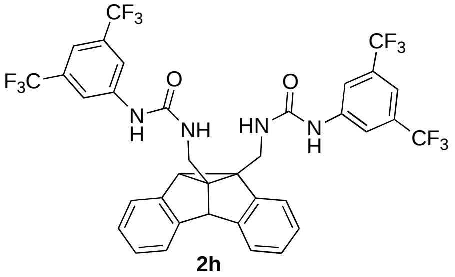
CF3
F3C
O
N
H
NH
HN
O
N
H
CF3
CF3
2h

in $(CD_3)_2CO$

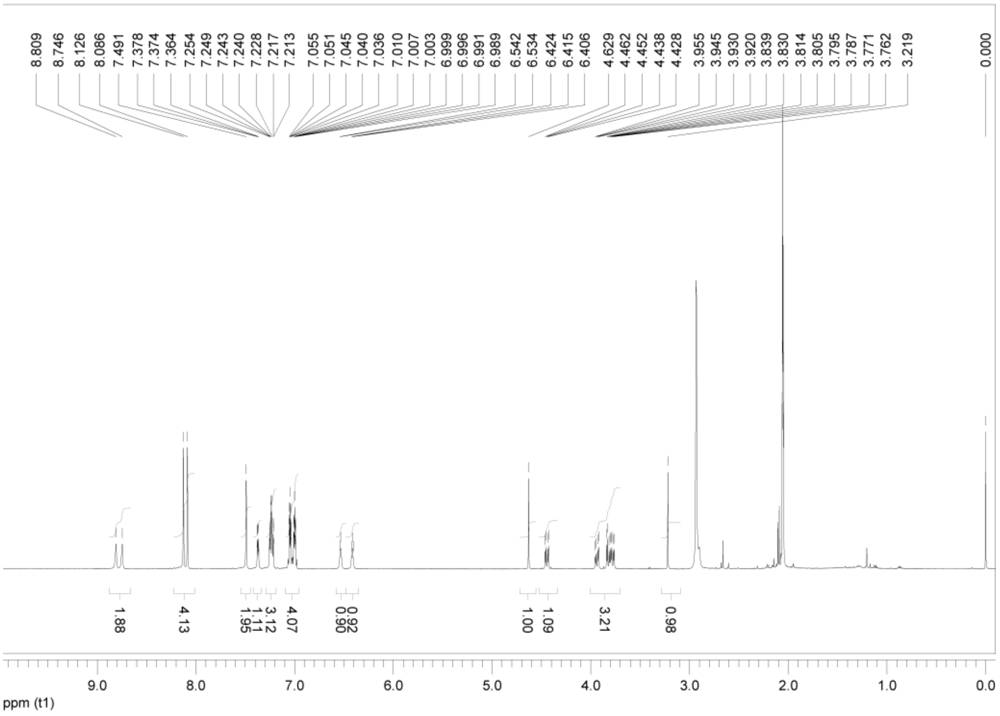
8.809
8.746
8.126
8.086
7.491
7.378
7.374
7.364
7.254
7.249
7.243
7.240
7.228
7.217
7.213
7.055
7.051
7.045
7.040
7.036
7.010
7.007
7.003
6.999
6.996
6.991
6.989
6.542
6.534
6.424
6.415
6.406
4.629
4.462
4.452
4.438
4.428
3.955
3.945
3.930
3.920
3.839
3.830
3.814
3.805
3.795
3.787
3.771
3.762
3.219
0.000
1.88
4.13
1.95
1.11
3.12
4.07
0.90
0.92
1.00
1.09
3.21
0.98
9.0
8.0
7.0
6.0
5.0
4.0
3.0
2.0
1.0
0.0
ppm (t1)

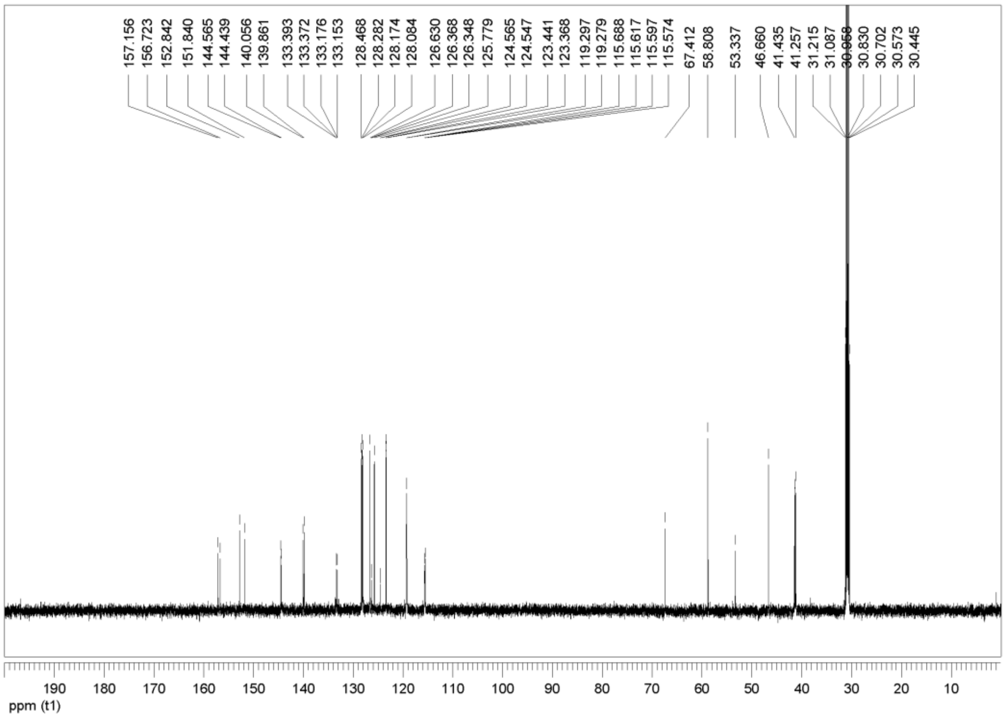
157.156
156.723
152.842
151.840
144.565
144.439
140.056
139.861
133.393
133.372
133.176
133.153
128.468
128.282
128.174
128.084
126.630
126.368
126.348
125.779
124.565
124.547
123.441
123.368
119.297
119.279
115.688
115.617
115.597
115.574
67.412
58.808
53.337
46.660
41.435
41.257
31.215
31.087
30.958
30.830
30.702
30.573
30.445
190
180
170
160
150
140
130
120
110
100
90
80
70
60
50
40
30
20
10
ppm (t1)

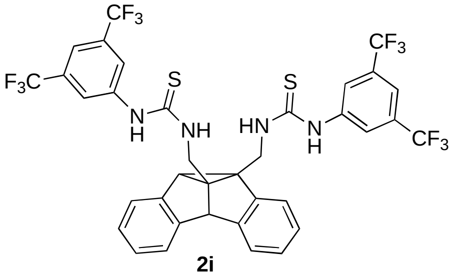
CF3
F3C
S
N
H
NH
HN
S
N
H
CF3
CF3

2i

in $(CD_3)_2CO$

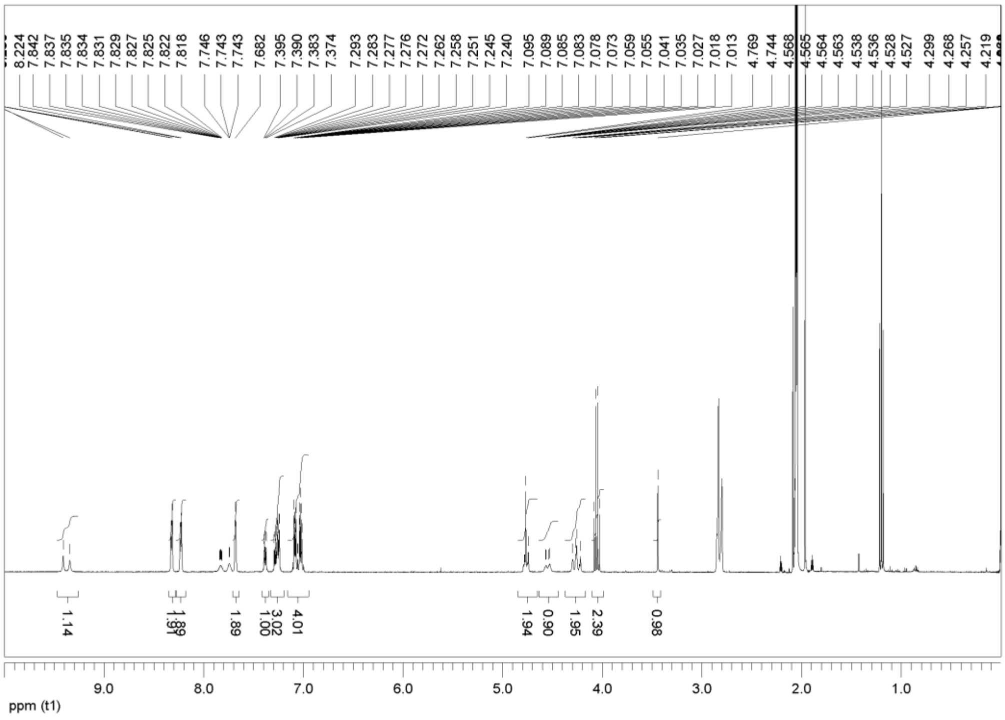
8.224
7.842
7.837
7.835
7.834
7.831
7.829
7.827
7.825
7.822
7.818
7.746
7.743
7.743
7.682
7.395
7.390
7.383
7.374
7.293
7.283
7.277
7.276
7.272
7.262
7.258
7.251
7.245
7.240
7.095
7.089
7.085
7.083
7.078
7.073
7.059
7.055
7.041
7.035
7.027
7.018
7.013
4.769
4.744
4.568
4.565
4.564
4.563
4.538
4.536
4.528
4.527
4.299
4.268
4.257
4.219
1.14
1.89
1.97
1.89
1.00
3.02
4.01
1.94
0.90
1.95
2.39
0.98
9.0
8.0
7.0
6.0
5.0
4.0
3.0
2.0
1.0
ppm (t1)

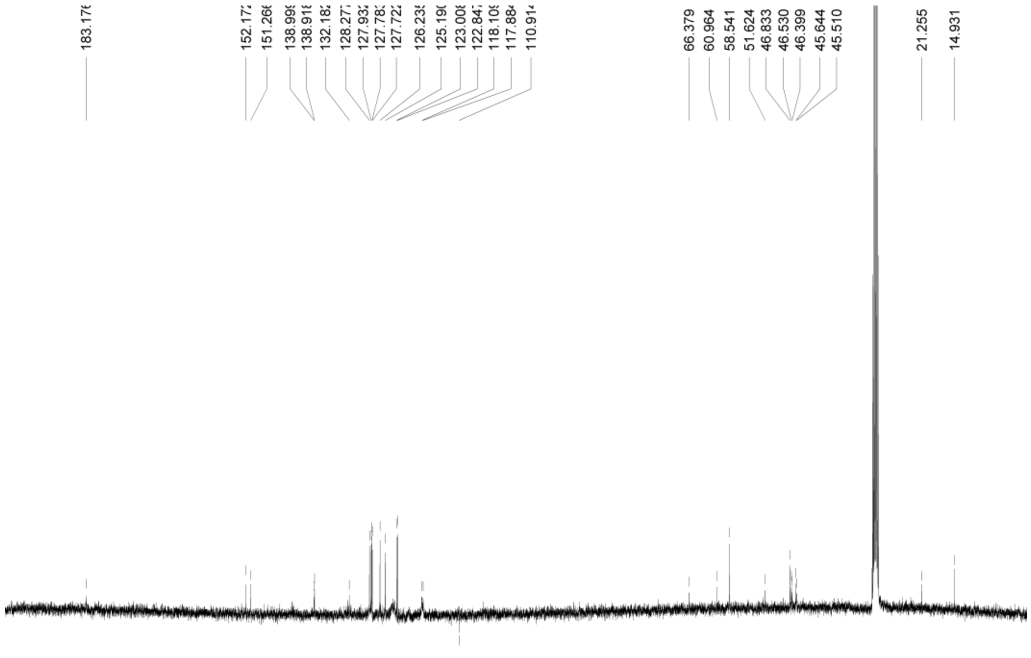
66.379
60.964
58.541
51.624
46.833
46.530
46.399
45.644
45.510
21.255
14.931
150
100
50

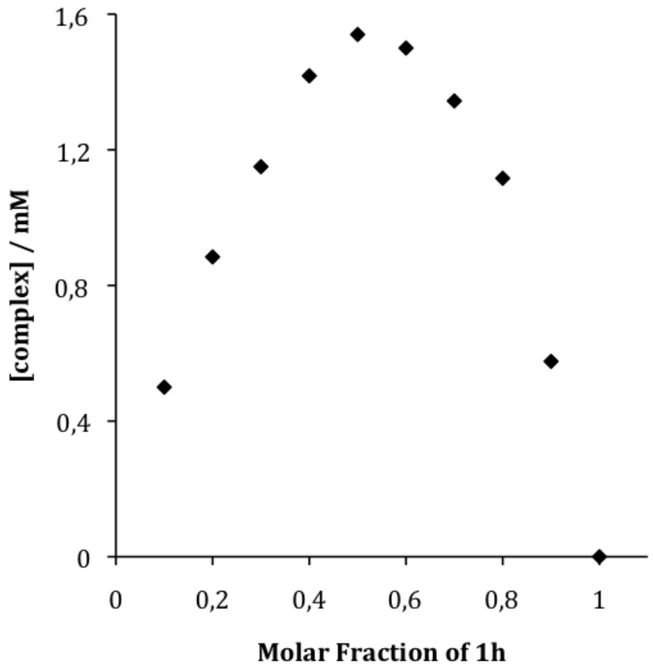

Figure S1: Job plot of **1h** with tetrabutylammonium (*S*)-mandelate (**SMD**) in $(CD_3)_2SO$, 400 MHz; determined from the ^1H NMR spectroscopic shift of the ar-NH proton at different molar fractions of **1h** (see following ^1H NMR spectra; overall concentration [**1h**] + [**SMD**] = 5.0 mM).

^{1}H NMR spectra of 1h with SMD at different molar ratio (1h:SMD from 10:0 to 1:9)

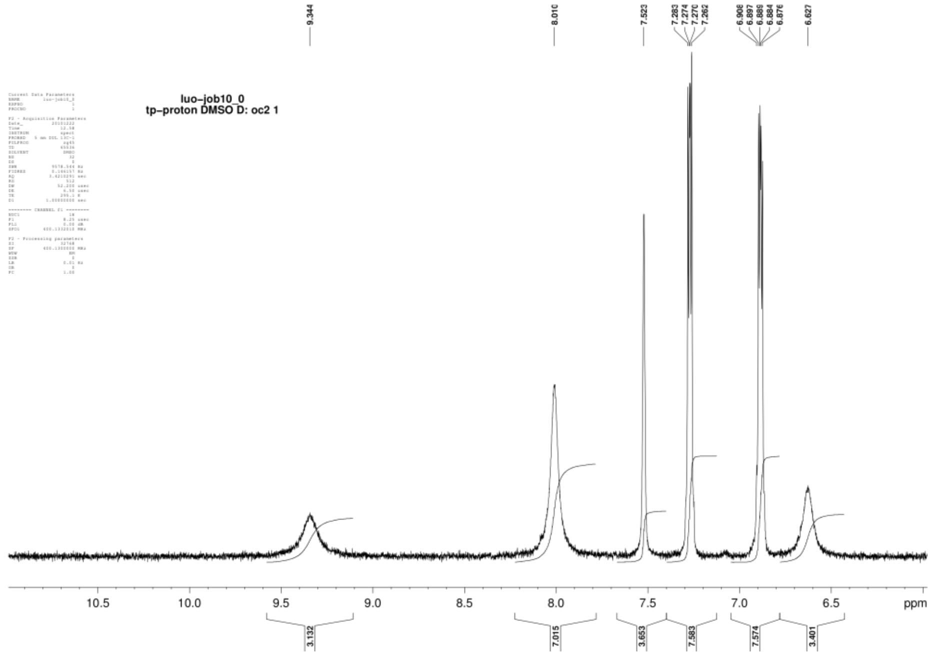

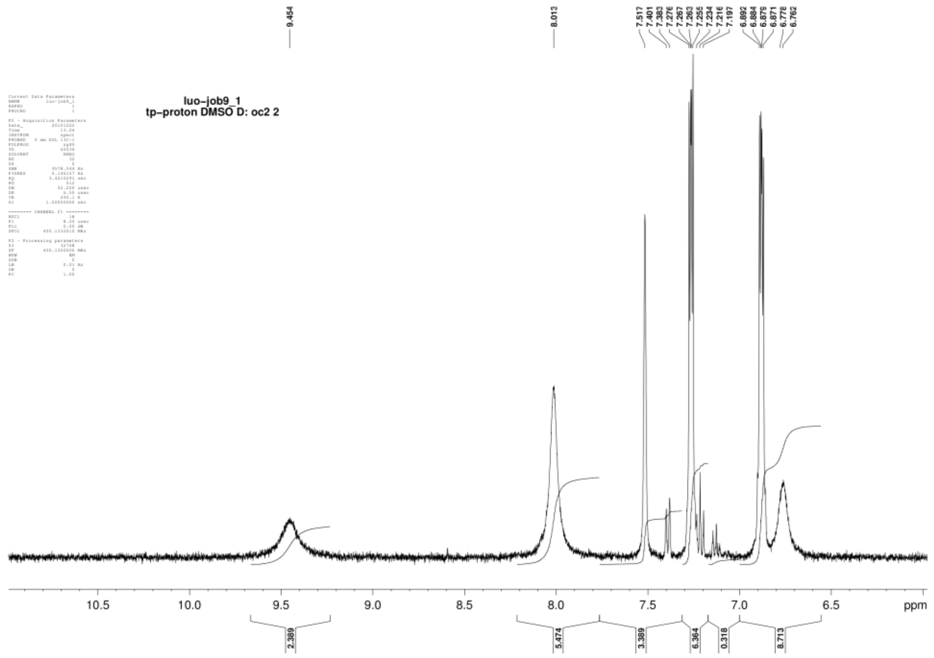

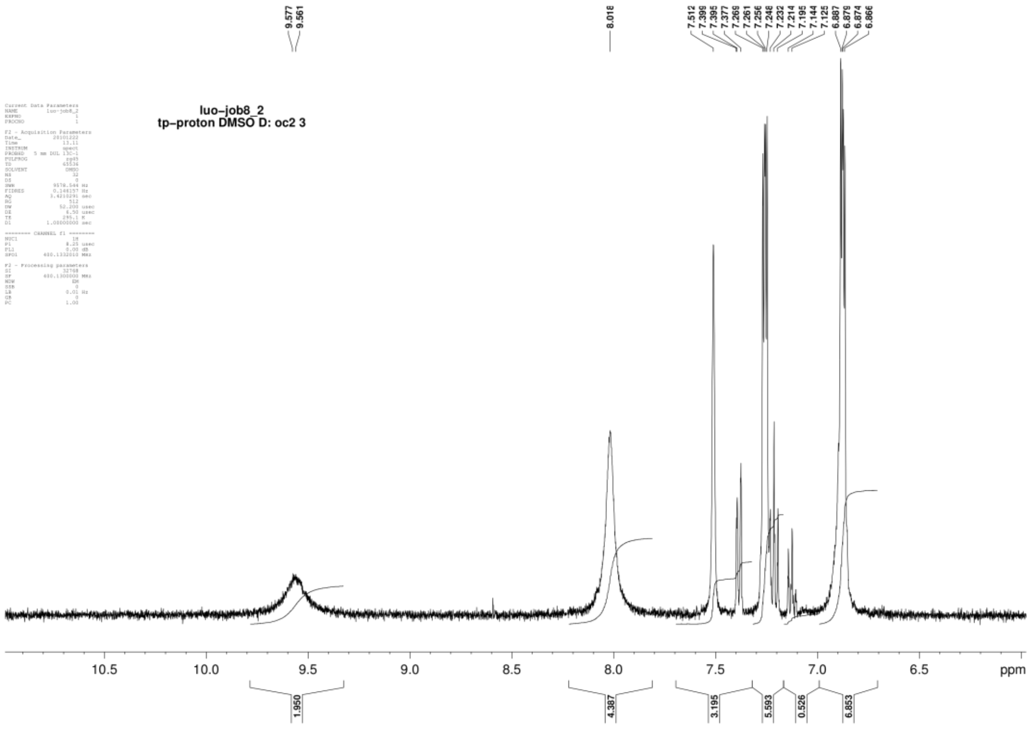

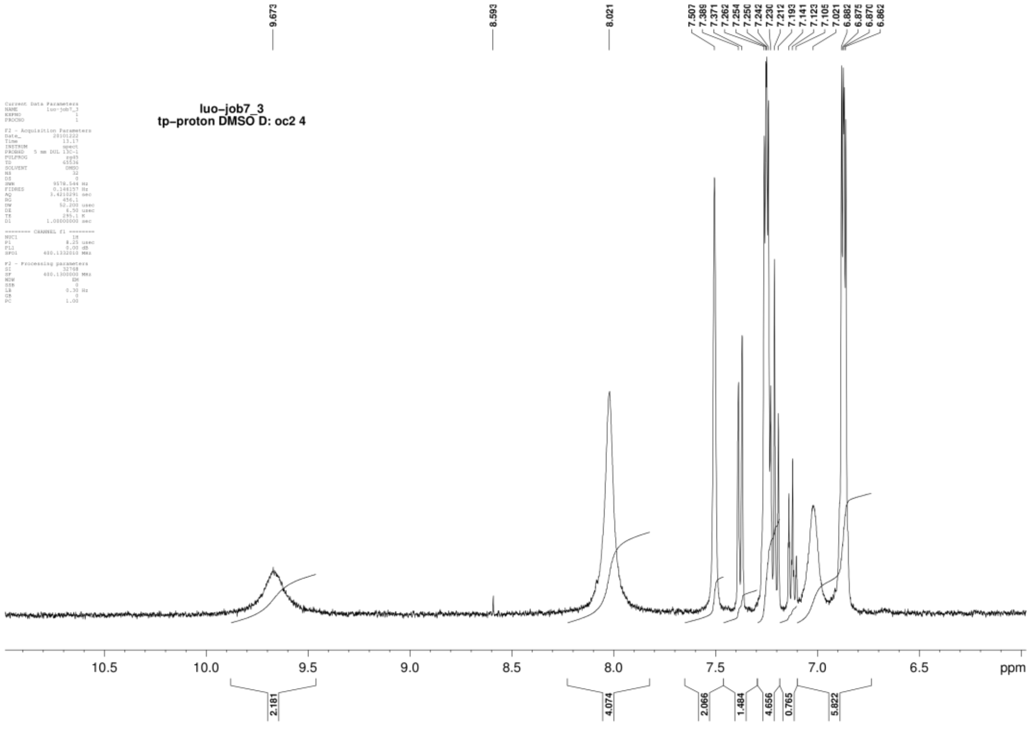

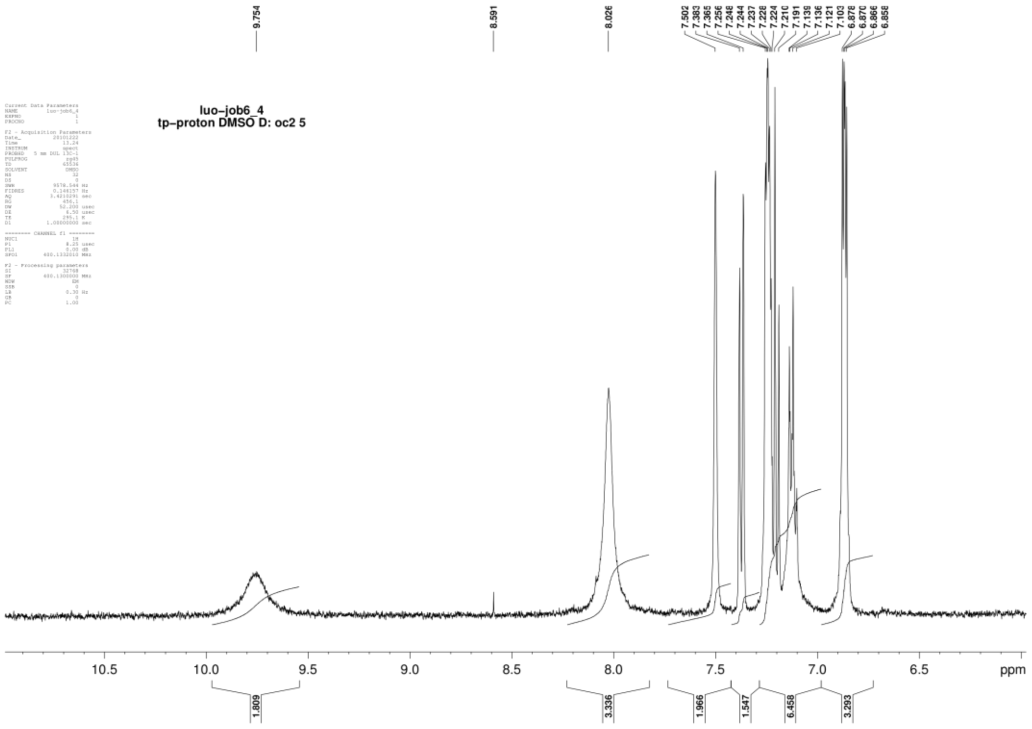
luo–job6_4
tp–proton DMSO D: oc2 5
ppm

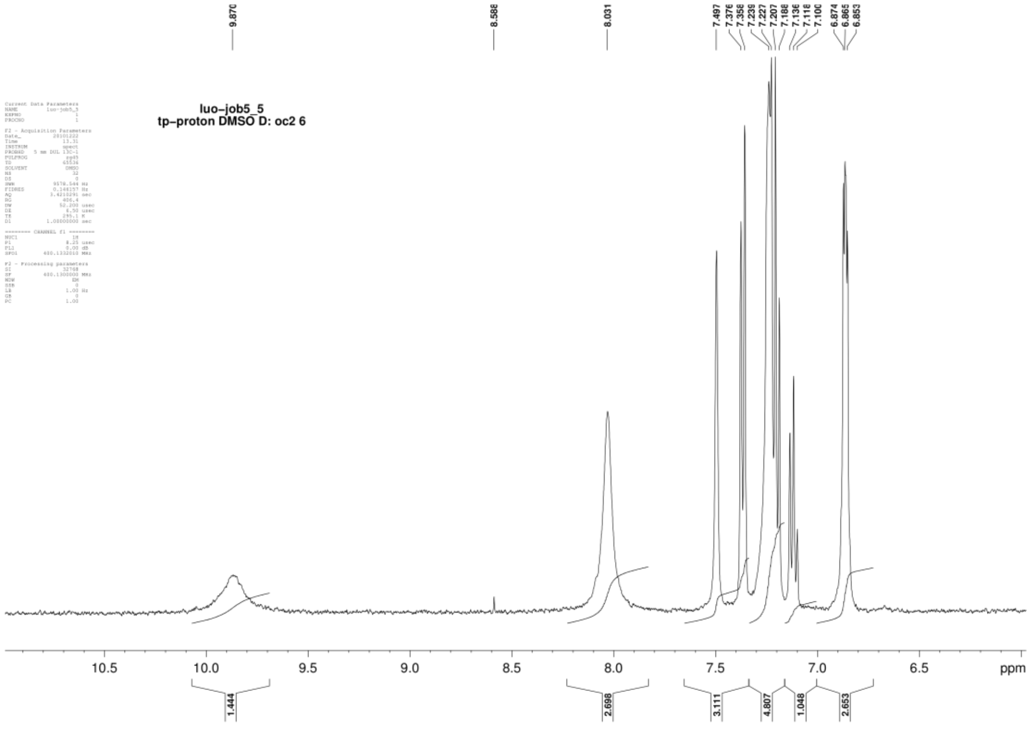
luo–job5_5
tp–proton DMSO D: oc2 6
ppm

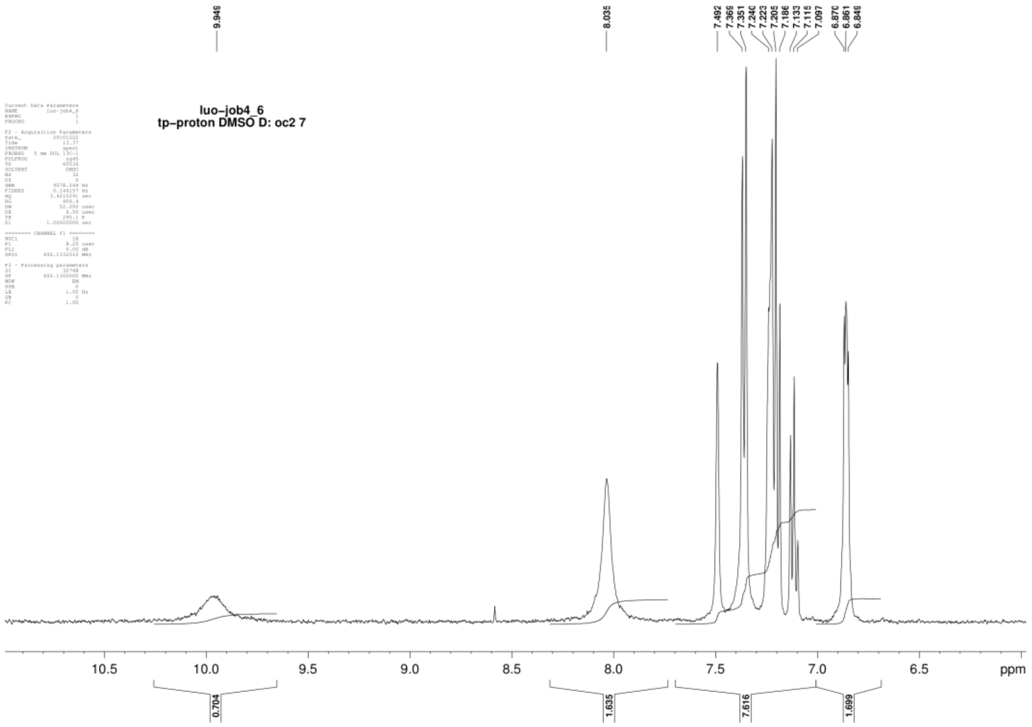
luo–job4_6
tp–proton DMSO D: oc2 7
9.949
8.035
7.492
7.369
7.351
7.240
7.223
7.205
7.186
7.133
7.115
7.097
6.870
6.861
6.849
10.5
10.0
9.5
9.0
8.5
8.0
7.5
7.0
6.5
ppm
0.704
1.635
7.616
1.699

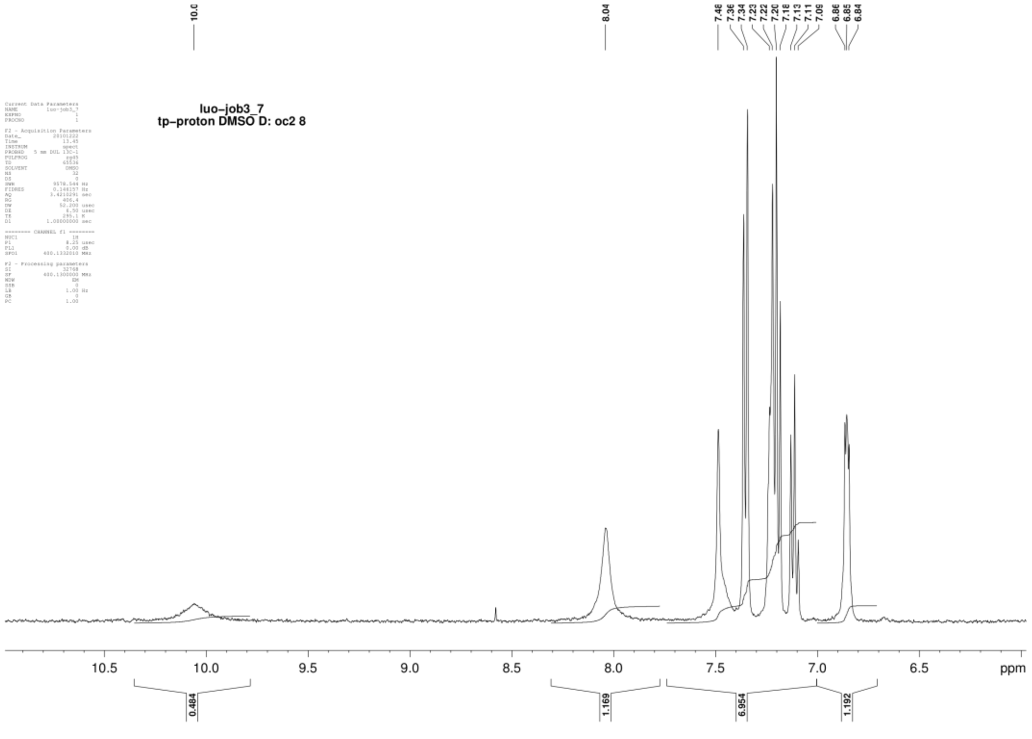
luo–job3_7
tp–proton DMSO D: oc2 8
8.04
10.5
10.0
9.5
9.0
8.5
8.0
7.5
7.0
6.5
ppm
0.484
1.169
6.954
1.192

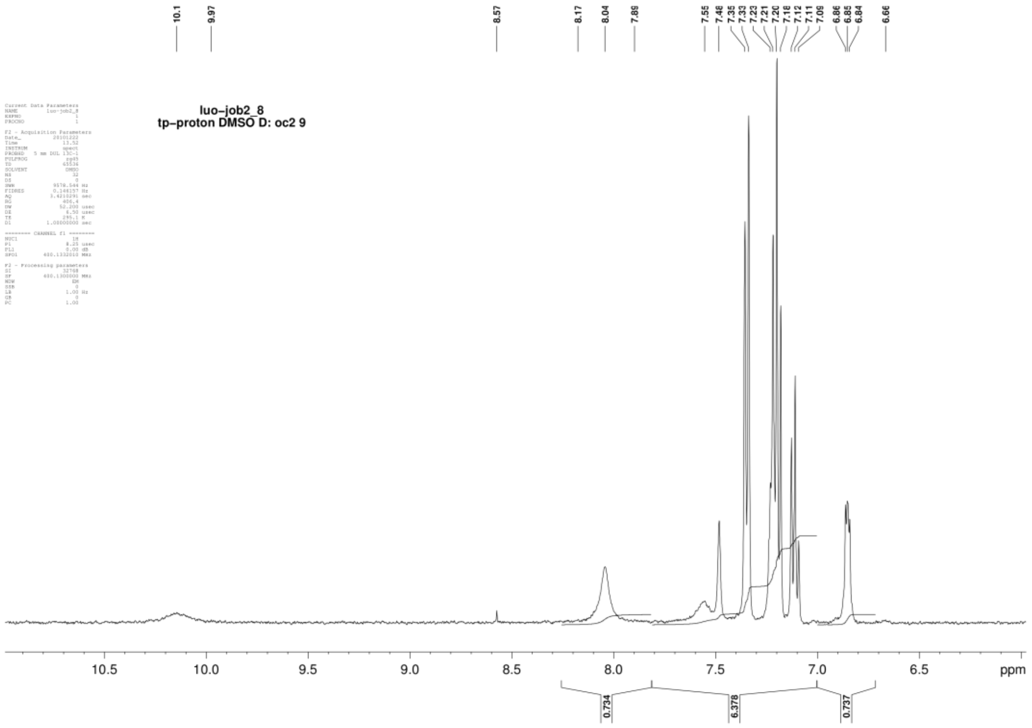
luo–job2_8
tp–proton DMSO D: oc2 9
ppm

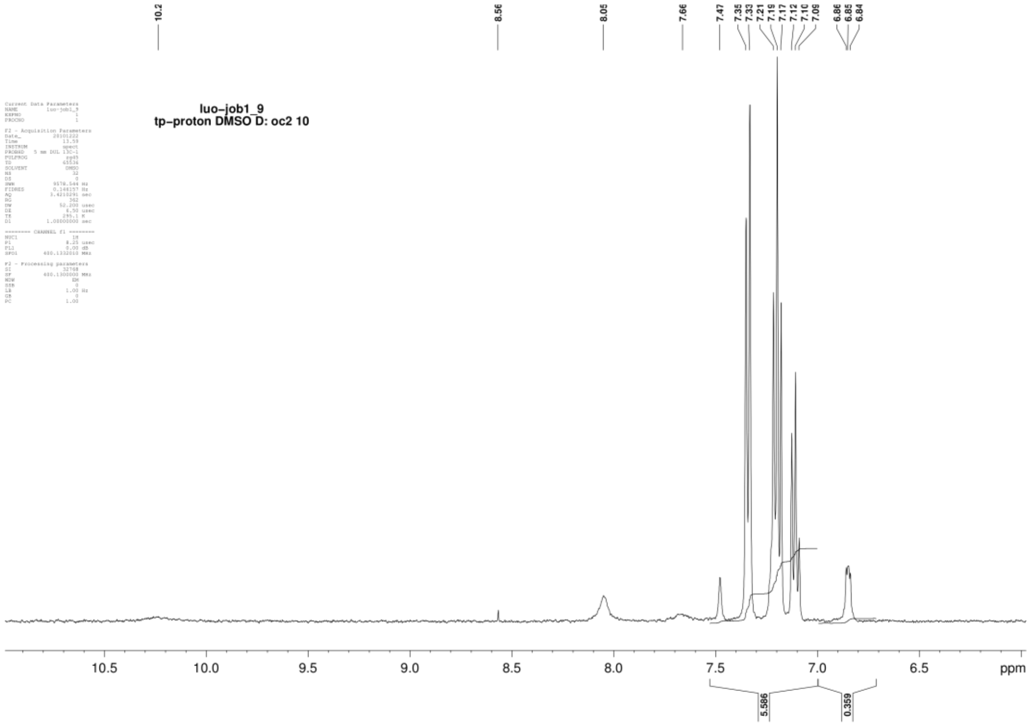
luo–job1_9
tp–proton DMSO D: oc2 10
ppm

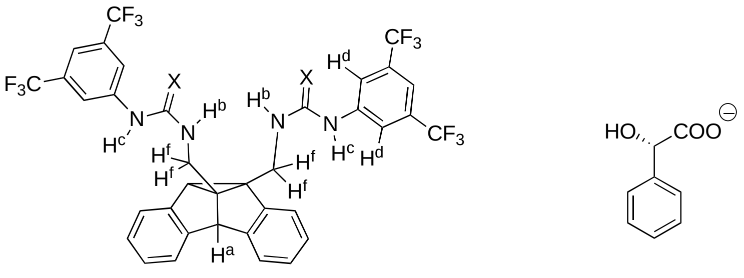

2h: X = O
2i : X = S

SMD

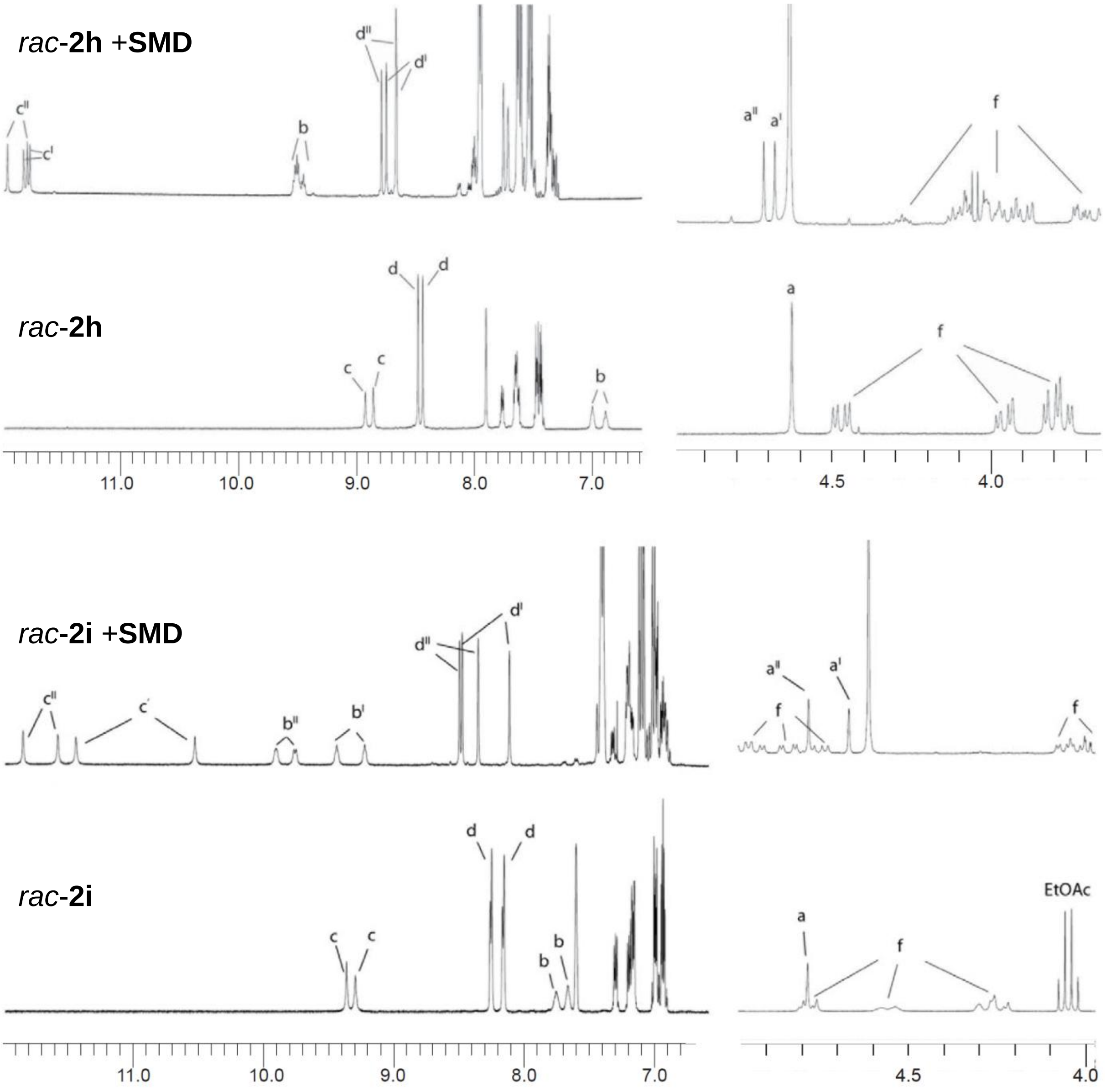

Figure S2: Tetrabutylammonium (*S*)-mandelate (**SMD**) as chiral shift reagent for the dibenzosemibullvalene derivatives **2h** and **2i**, in $(CD_3)_2CO$, 400 MHz.
